# Sertoli Cells Express Accommodation, Survival, and Immunoregulatory Factors When Exposed to Normal Human Serum

**DOI:** 10.3390/biomedicines11061650

**Published:** 2023-06-06

**Authors:** Rachel L. Washburn, Dalia Martinez-Marin, Tyler Sniegowski, Ksenija Korać, Alexis R. Rodriguez, Jonathan M. Miranda, Beverly S. Chilton, Robert K. Bright, Kevin Pruitt, Yangzom D. Bhutia, Jannette M. Dufour

**Affiliations:** 1 Department of Cell Biology and Biochemistry, School of Medicine, Texas Tech University Health Sciences Center, Lubbock, TX 79424, USA; rachel.washburn@ttuhsc.edu (R.L.W.); dalia.martinez-marin@ttuhsc.edu (D.M.-M.); tyler.sniegowski@ttuhsc.edu (T.S.); ksenija.korac@ttuhsc.edu (K.K.); alexis.r.rodriguez@ttuhsc.edu (A.R.R.); jonathan.m.miranda@ttuhsc.edu (J.M.M.); beverly.chilton@ttuhsc.edu (B.S.C.); yangzom.d.bhutia@ttuhsc.edu (Y.D.B.); 2 Department of Immunology and Molecular Microbiology, School of Medicine, Texas Tech University Health Sciences Center, Lubbock, TX 79424, USA; robert.bright@ttuhsc.edu (R.K.B.); kevin.pruitt@ttuhsc.edu (K.P.)

**Keywords:** Sertoli cells, xenotransplantation, complement, immune regulation

## Abstract

Transplantation is a clinical procedure that treats a variety of diseases yet is unattainable for many patients due to a nationwide organ shortage and the harsh side effects of chronic immune suppression. Xenografted pig organs are an attractive alternative to traditional allografts and would provide an endless supply of transplantable tissue, but transplants risk rejection by the recipient’s immune system. An essential component of the rejection immune response is the complement system. Sertoli cells, an immunoregulatory testicular cell, survive complement as xenografts long term without any immune suppressants. We hypothesized that exposure to the xenogeneic complement influences Sertoli cell gene expression of other accommodation factors that contribute to their survival; thus, the purpose of this study was to describe these potential changes in gene expression. RNA sequencing of baseline neonatal pig Sertoli cells (NPSC) as compared to NPSC after exposure to normal human serum (NHS, containing complement) revealed 62 significantly differentially expressed genes (DEG) that affect over 30 pathways involved in immune regulation, cell survival, and transplant accommodation. Twelve genes of interest were selected for further study, and Sertoli cell protein expression of CCL2 and the accommodation factor A20 were confirmed for the first time. Functional pathway analyses were conducted in NPSC and three biological clusters were revealed as being considerably affected by NHS exposure: innate immune signaling, cytokine signaling, and T cell regulation. Better understanding of the interaction of Sertoli cells with complement in a xenograft environment may reveal the mechanisms behind immune-privileged systems to increase graft viability.

## 1. Introduction

Though transplantation is important in treating a wide range of illnesses and disorders, it remains unattainable for many patients on the organ waiting list as there is not enough transplantable tissue available to meet the need. A feasible solution is to use pigs as xenograft donors, providing an unlimited supply of organs and tissues [1]. Recently, a patient received a transgenic pig heart to treat end-stage heart failure [2]. The pig donor was genetically modified to knock out the expression of three xenoantigens (XNA) and to express various survival and immune suppressive factors. The patient also underwent an extensive and experimental immune suppression regimen to prolong xenograft survival [2]. The heart functioned for two months until the patient’s death, marking a milestone in the transplantation field [2]. Though utilizing xenografted pig tissue would effectively solve the organ shortage, this clinical study illustrates a risk of allotransplantation and xenotransplantation—graft rejection.

Curiously, Sertoli cells (SC) have been shown to survive as both allografts and xenografts long-term, 100+ days [3] or 90+ days [4] post-transplantation, respectively, without any immunosuppressant treatment. SC are physiologically located in the seminiferous tubules of the testis where they protect germ cells from autoimmune destruction, and transplanted SC have also been shown to create this immune-privileged environment outside the testis to increase the survival of co-grafted cells [5,6,7].

As prolonged complement activation is a leading cause of chronic kidney graft rejection due to vascular occlusion by antibody-mediated complement complexes and ischemia [8,9], this study focuses on the interaction of SC and complement in xenotransplantation. Neonatal pig SC (NPSC) exposed in vitro to either heat-inactivated normal human serum (NHS) and rabbit complement or non-heat-inactivated NHS survive above media controls, while the survival of neonatal pig islets (NPI) and pig aortic endothelial cells (PAEC) was significantly decreased [10,11]. PAEC cultured in pig SC conditioned media (SCCM) experienced significantly elevated survival (two-fold increase) after exposure to human complement serum, implying that pig SC produce secreted factor(s) that confer protection to complement-killed cells [11].

Recently, complement has been indicted in the regulation and activation of immune cells, particularly T cells and antigen presenting cells (APC) [12,13]. The anaphylatoxins C3a and C5a have been shown to activate signaling pathways important for quiescence and survival, activation and expansion, and suppression of T cell and APC responses [13,14]. The activation complement component C1q has also been implicated in immune cell activation and function after binding its receptor C1q binding protein (C1QBP, C1q receptor, gC1qR) on mitochondria to foster mitochondrial fitness, cell survival, and proliferative capacity [15,16]. Evidently, complement components participate in shaping the immune environment by modulating APC and T cell regulation.

Taking this into account, we hypothesized that 1.5 h exposure of SC to xenogeneic complement influences differential expression of survival, accommodation, and immunoregulatory genes that are important to their survival as xenografts. RNA sequencing identified 62 significantly differentially expressed genes (DEG) between baseline and complement-exposed cells, many of which are involved in cell survival, proliferation, accommodation, and immune regulation. The qPCR of 12 genes of interest (GOI) confirmed statistically significant changes in 9 of these genes. Protein expression of the GOI A20 and CCL2 was confirmed for the first time in SC. Gene ontology analyses indicated that the cellular responses most affected by DEG are involved in cell survival to stress, immune regulation, inflammation modulation, proliferation, and angiogenesis. The top pathways containing the most DEG followed this same trend, with the majority involved in innate immune responses, cytokine responses, and T cell regulation. Overall, the purpose of this study was to describe the changes in gene expression after NPSC exposure to NHS after 90 min. Determining whether complement signaling on SC leads to the expression of factors that create and maintain an immunomodulatory environment can be translated into focusing future research on the therapeutic potential of these genes in prolonging graft survival and reducing inflammation.

## 2. Materials and Methods

### 2.1. Animals

Testes were collected from three-to-five-day-old Duroc-Landrace pigs from the Texas Tech University New Deal Swine Unit. All procedures were in adherence to the approved Institute for Laboratory Animal Research Care, Use of Laboratory Animals, Texas Tech University Health Sciences Center Institutional Animal Care and Use Committee guidelines and protocols of the National Institutes of Health (IACUC protocol 05019).

### 2.2. Sertoli Cell Isolation

Testes were collected from 3 litters of 4 male pigs on different farrowing dates for n = 24 testes, n = 12 pigs, and n = 3 litters. Testes from each litter were sterilized in 70% ethanol twice for 30 s each and stored in sterile HBSS on ice for transit. Testis were chopped vigorously by hand in a sterile hood for roughly 10 min, and then collagenase and trypsin digestions and filtration were used to isolate NPSC from testicular tissue, as previously described [4]. NPSC were cultured on 150 mm tissue culture plates (Corning Inc., Corning, NJ, USA) using Dulbeco’s modified eagle medium (DMEM, Sigma-Aldrich, Burlington, MA, USA) media containing 10% fetal bovine serum (FBS) as described below.

### 2.3. RNA Sequencing

Three million NPSC were cultured as a monolayer per 100 mm tissue culture plate (Corning Ing, Corning, NJ, USA) in 10 mL DMEM + 10% FBS overnight at 37 °C and 5% CO_2_. Then, 5 mL of media was removed from each plate and either 5 mL DMEM (baseline NPSC plates, n = 3) or 5 mL of pooled AB NHS with complement preserved (complement NPSC plates, n = 3) was added to replicate the conditions of the NHS cytotoxicity assay used frequently in our lab [10]. After incubation for 1.5 h [10,11], media and media containing NHS was removed and plates were gently rinsed twice with phosphate buffered saline (PBS, pH 7.4). PBS was added to each plate, and cells were carefully scraped and placed in sterile 50 mL conical tubes. Tubes were centrifuged for 5 min at 800 RPM, then supernatant was removed, and cells were rinsed twice more with PBS. To prepare cells for RNA sequencing, baseline NPSC and NPSC + complement were lysed with 1 mL of Trizol^®^ reagent (Ambion by Life Technologies, Carlsbad, CA, USA). Samples were stored at −80 °C in Trizol^®^ until all samples were collected (n = 3 of each condition). Then, samples were packaged in dry ice and shipped to GENEWIZ (Azenta Life Sciences, South Plainfield, NJ, USA) for RNA isolation, RNA sequencing, and data processing.

GENEWIZ from Azenta Life Sciences conducted next generation sequencing on all samples. The RNA sequencing service prepared the library for standard and strand-specific RNA, small RNA, and ultra-low input RNA. For standard and strand-specific service, the target RNAs is mRNA, which undergoes poly(A) selection, and mRNA + long non-coding RNA (lncRNA), which undergoes ribosomal RNA (rRNA) depletion. For small RNA sequencing service, target RNA are small RNAs including microRNA (miRNA), small interfering (siRNA), and piwi-interacting RNA (piRNA), which were selected by size fractionation with adaptor ligation to 5′ phosphatase. For ultra-low input RNA sequencing service, mRNA was selected by poly(A) selection with enrichment for full-length transcripts. Sequencing was performed on the Illumina^®^ NovaSeq™ or HiSeq^®^ platforms with the following conditions 2 × 150 base pairs (bp) configuration, 20–30 million read depth, and a guaranteed data quality of ≥80% bases with at least Q30. Data analysis included trimming, mapping, DEG, small RNA discovery, and novel transcript discovery. Sample quality control report, aligned data, hit counts, DEG results, gene ontology (GO) enrichment analysis, and differential splicing analysis were included as deliverables.

### 2.4. PCR for Genes of Interest

The Purelink™ RNA Mini Kit (Invitrogen, Carlsbad, CA, USA) was used to isolate total RNA for qPCR. Briefly, 3 × 10^6^ NPSC were lysed in 0.6 mL lysis buffer with 1% 2-mercaptoethanol (BioRad Laboratories, Hercules, CA, USA) and passed 10 times through a sterile 21-gage needle. A series of washes and filtrations were performed per the manufacturer’s instructions, and the RNA was suspended in nuclease-free water and stored at −80 °C until ready for cDNA conversion and qPCR. RNA was quantified using a nanodrop (Nanodrop one, Thermo Fisher Scientific, Waltham, MA, USA). Then, the High-Capacity cDNA Reverse Transcriptase Kit with RNase Inhibitor (Applied Biosciences, Waltham, MA, USA) and the ProFlex PCR System thermocycler (Applied Biosciences, Waltham, MA, USA) were used to reverse transcribe total RNA into cDNA.

qPCR was conducted using the QuantStudio™ 3 Real Time PCR system (Applied Biosystems by Thermo Fischer Scientific, Waltham, MA, USA) with iTaq Universal SYBR^®^ Green Supermix (Bio Rad Laboratories, Inc., Hercules, CA, USA). Primers are contained in Table S1. Gene expression was normalized to an endogenous control (GAPDH).

### 2.5. Protein Collection and Quantification by Western Blot and ELISA

Total cellular proteins (n ≥ 3) were isolated from baseline and complement SC through cell lysis using radioimmunoprecipitation assay buffer consisting of tris-hydrochloric acid (20 mM, pH 7.5), sodium chloride (150 mM), 1% nonyl-phenyl polyethylene 40, 0.1% sodium dodecyl sulfate, 1% deoxycholic acid, sodium fluoride (5 mM), ethylenediaminetetraacetic acid (1 mM). Proteins were then put on ice for 30 min and centrifuged at 12,000× *g* for 5 min. Protein concentrations were determined by the Bradford assay (BioRad, Hercules, CA, USA) with BSA as the standard [17].

Western blot was used to quantify A20 (*TNFAIP3*) protein levels in baseline and complement NPSC. Actin was used to control for loading with mouse anti-actin primary (1:2000, Chemicon International, Temecular, CA, USA) and anti-mouse secondary to control for loading. A 10% SDS-polyacrylamide gel and electrophoresis were used to separate cellular lysates. Proteins were transferred to immobilon-P membranes, which were blocked with 5% milk, then incubated with A20 primary antibody for six hours (1:1000, Proteintech, Rosemont, IL, USA). Next, membranes were further incubated with goat-anti-rabbit IgG (H+L) secondary antibody (1:40,000, Invitrogen, Waltham, MA, USA) for 2 h. Before imaging, membranes were incubated in SuperSignal™ West Pico PLUS Chemiluminescent Substrate (Thermo Fisher Scientific, Waltham, MA, USA) and imaged using the Azure 300 western blot imaging system (Azure Biosystems, Dublin, CA, USA). A20 protein levels were normalized to actin and relative protein levels were calculated between baseline and complement NPSC.

NPSC conditioned media (SCCM) was collected as follows: 0.2 × 10^6^ NPSC were plated on 24-well tissue culture plates in 1 mL of DMEM + 10% FBS for 18–24 h. Then, 500 uL of media was removed per well and either 500 mL of DMEM + 10% FBS or 500 uL of NHS containing complement was added per well. After incubation for 1.5 h, all media was removed, then centrifuged at 1000× *g* for 15 min to remove cell debris.

Secreted protein levels of CCL2 were quantified in SCCM or media-only controls by ELISA assay (Invitrogen). ELISA was performed per manufacturer’s instructions. Briefly, the standards or diluted samples (1:2 dilution of SCCM) were added per well (antibody pre-coated). Next, biotinylated detector antibody was added, followed by avidin-horse radish peroxidase and then 3,3′,5,5′-tetramethylbenzidine (TMB) substrate. Manufacturer recommended washes and incubations were performed per manufacturer protocols. Stop solution was added and the plate was read at an optical density of 450 nm O.D.

### 2.6. Bioinformatic Analyses

Bioinformatic and statistical analyses of initial RNA sequencing data were performed by GENEWIZ from Azenta Life Sciences (South Plainfield, NJ, USA). Read count distributions in libraries were assessed before normalization and after normalization. Normalization of read counts were performed to adjust for factors such as sequencing yield variations between samples and were used to ascertain DEG. To identify any samples not representative of their group and to prevent issues with analyses quality, data quality assessments were conducted. To examine the similarity and difference of samples to each other and their relationship to experimental design expectations, the Euclidean distance between samples was calculated. A principal component analysis (PCA) was also conducted to further analyze sample similarity via distance matrix, and samples were projected by their first two principal components to a 2D plane to allow for visualization of the overall effect of experimental covariates and sample batch effects. A comparison of gene expression between NPSC at baseline (baseline NPSC) and NPSC after complement exposure (complement NPSC, comp) was performed using DESeq2 and the Wald test to generate *p*-value, adjusted *p*-value (*p*-adj, and log2 fold changes (LFC). *p*-adj was calculated by dividing the false discovery rate by q-value to control for the rate of any false positives. DEGs were identified as genes with *p*-adj < 0.05 and absolute log2FC > 1. To identify potential co-regulated genes at baseline and after complement exposure, a bi-clustering heatmap was constructed (Figure S1A), allowing for visualization of the top 40 DEG expression profiles. A heatmap was created to visualize the relationship and co-regulated genes between the top 30 DEG using *p*-values and log2 transformed expression (Figure S1B). A volcano plot was used to visualize global transcriptional changes between baseline and complement NPSC gene expression (Figure S1C). A pathway plot was constructed to display the iPathwayGuide Impact Analysis. This analysis includes over-representation of DEG in a pathway and pathway topology (Figure S1D). A dendrogram was constructed to visualize the relationships of the various DEG between GO terms, which was used to select genes of interest (Figure S1E).

Significantly DEG were then clustered by GO, which was tested using Fisher’s exact test (GeneSCF v1.1-p2). Further data (significantly impacted pathways, biological processes, molecular interactions, miRNAs, SNPs, etc.) were analyzed using Advaita Bio iPathwayGuide (Ann Arbor, MI, USA) [18,19]. Pathway diagrams include the measured expression change for each gene as determined by log fold change (LFC) from the KEGG pathway. Total accumulation was calculated by using documented interactions between DEG and other genes, then determining propagation, the effect of changes in downstream gene expression. Each pathway diagram displays the total perturbation of DEG effects on the pathway. Perturbation is a combination of the measured LFC and total accumulation of the genes. The change of expression measurement is based on the selected KEGG pathway + total accumulation and is representative of the propagation of the total effect of changes in gene expression of upstream and downstream genes based on documented interaction already established.

### 2.7. Statistical Analyses

All values are expressed as means ± standard error of mean, one-way ANOVA, or unpaired *t*-test per row and individual variances computed for each comparison. Statistical significance between groups was set at *p* < 0.05. Statistical analyses were performed using GraphPad Prism9 software (Dotmatics, San Diego, CA, USA).

## 3. Results

### 3.1. DEG Identified by RNA Sequencing

RNA sequencing data analyses revealed 62 significant DEG between baseline and complement NPSC (Table 1). Of these DEG, the expression of 53 were significantly increased with a LFC > 0.7 (Figure S1C, red dots; Table 1, red text) and 9 were significantly decreased with a LFC < −0.7 (Figure S1C, blue dots; Table 1 blue text). The gene for lysozyme (LYZ) was the most significantly decreased with −2.312 LFC (*p*-adj < 0.0001) and the gene for CCL20 was most significantly increased with 3.613 LFC (*p*-adj < 0.0001).

**Figure 1:**
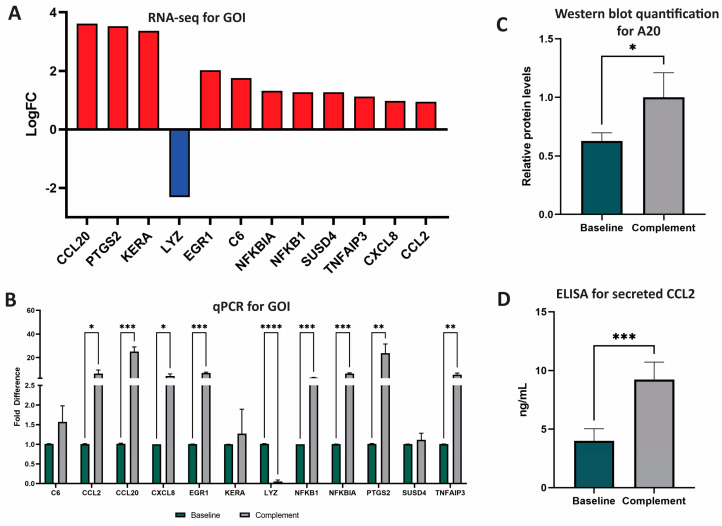
RNA and protein analyses of GOI after NPSC exposure to NHS. (**A**) Twelve genes of interest (GOI) were identified and selected based on LFC and number of pathways impacted. Advaita and GENEWIZ were used to identify DEG and GOI. DEG were determined with LFC ≥ 1 and *p*-adj ≤ 0.05. GOI include *CCL20*, *PTGS2*, *KERA*, *LYZ*, *EGR1*, *C6*, *NFKBIA*, *NFKB1*, *SUSD4*, *TNFAIP3*, *CXCL8*, and *CCL2.* (**B**) qPCR analyses of the top 12 DEG between baseline NPSC (black) and complement NPSC (white) identified nine GOI with significantly different expression after complement exposure: *CCL2*, *CCL20*, *CXCL8*, *EGR1*, *LYZ*, *NFKB1*, *NFKBIA*, *PTGS2*, and *TNFAIP3*. (**C**) Relative protein levels of A20 as quantified from western blot determined that baseline NPSC express A20 at 0.63 ± 0.04, while complement NPSC express A20 at 1.00 ± 0.12. (**D**) ELISA analysis was used to quantify protein expression and secretion of CCL2 by baseline NPSC (4.00 ± 0.52 ng/mL green bar) and complement NPSC (9.23 ± 0.61). Statistical significance was calculated by unpaired *t*-test. * *p* < 0.05. ** *p* < 0.01. *** *p*< 0.001. **** *p* < 0.0001.

From these 62 significant DEG, we selected 12 genes of interest (GOI) to analyze further (Figure 1). GOI were chosen based on complement-association, LFC, and involvement in the top five affected pathways. GOI chosen due to complement association were *C6* (cascade component) and *SUSD4* (inhibitor). GOI chosen by LFC were *KERA* (third highest, +3.371) and *LYZ* (lowest, −2.312) (Figure 1A). GOI chosen by pathway involvement were *NFKB1* (five pathways), *CCL2* (four pathways), *NFKBIA* (three pathways), *CXCL8* (three pathways), and *TNFAIP3* (three pathways). GOI chosen by LFC and pathway involvement were *CCL20* (highest LFC, +3.613, five pathways), *PTGS2* (second highest LFC, +3.529, three pathways), *EGR1* (in top 10 of LFC, +2.018, one pathway).

### 3.2. Gene Expression Analysis

qPCR was used to validate RNA sequencing results and to quantify RNA expression by GOI. Of the 12 GOI, the expression of 8 were significantly increased (*CCL2*, *CCL20*, *CXCL8*, *EGR1*, *NFKB1*, *NFLBIA*, *PTGS2*, *TNFAIP3*) and expression of 1 was significantly decreased (*LYZ*) (Figure 1B, Tables S2 and S3). Overall, qPCR analyses identified nine total GOI with statistically significant changes in expression after NPSC exposure to the human complement.

### 3.3. Protein Expression Analysis of A20 (TNFAIP3) and CCL2

A20, the protein encoded by the gene *TNFAIP3*, has recently been identified as an important factor in the development of graft accommodation [22]. Since *TNFAIP3* (A20 protein) is a DEG and is involved in many of the top-affected pathways, we confirmed the expression of this marker by Western blot for the first time in SC. The relative expression of A20 in baseline NPSC was 0.63 ± 0.04 relative protein expression, which was significantly elevated (*p* = 0.04) in complement NPSC at 1.00 ± 0.12 relative protein levels (Figure 1C).

CCL2 is an immune cytokine that has both anti-inflammatory and inflammatory functions. CCL2 is associated with immune tolerance and recruitment of suppressive immune cells such as myeloid derived suppressor cells (MDSC) and regulatory T cells (Tregs) in cancer [23,24,25]; these cells are also implicated in graft survival [26]. CCL2 has also been shown to have proinflammatory effects, so the regulation of this cytokine is important in transplantation [24]. Since CCL2 was one of the top DEG and has not yet been established as being produced by pig SC, secretion of CCL2 by baseline NPSC was confirmed and quantified by ELISA assay. NPSC conditioned media contained significantly (*p* = 0.0003) elevated levels of CCL2 at 10.416 ± 0.56 ng/mL (Figure 1D).

### 3.4. GO and Pathway Analyses

**Figure 2:**
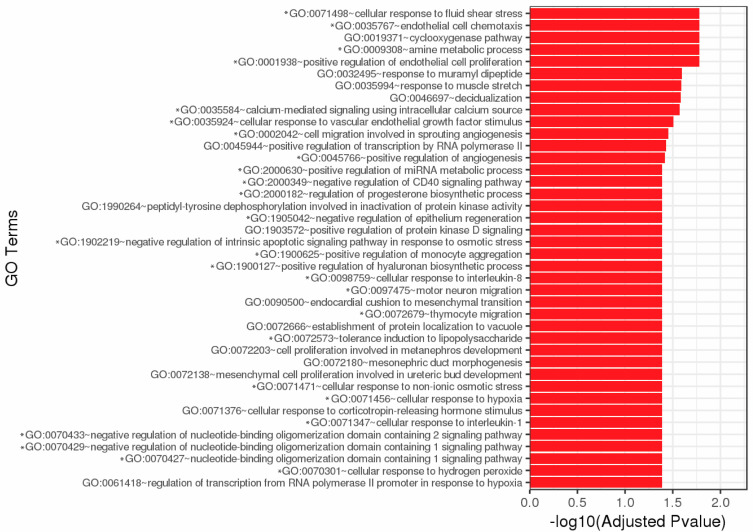
Top 40 GO enrichment. Significantly differentially expressed genes were clustered by their GO and the enrichment of GO terms was tested using Fisher exact test. These GO terms are significantly enriched with *p*-adj < 0.05 in DEG sets. Asterisks (*) denote GO terms involved in immunity, cell survival, and stress responses. Figure created by GENEWIZ.

Total GO consists of 40 cellular processes affected by complement exposure including chemotaxis, metabolic processes, proliferation, growth factor stimulus, migration, angiogenesis, regulation of immune responses (CD40, IL-1, IL-8, monocytes, etc.), and survival (Figure 2). Over 60 pathways were significantly affected by DEG resulting from complement exposure (Figure S1D,E). Twenty-nine DEG are immune-related genes (Table 2). Many of these pathways have various DEG in common (Figure S1E, Table 3). Most affected pathways are involved in immune regulation, inflammation, cytokine signaling, survival, and proliferation pathways.

### 3.5. Functional Pathway Analyses of Immune-Related Genes

We compiled a list of immune-related genes using the Immunome Knowledge Database [20] and InnateDB [21] online databases to focus on pathways related to immune response (Table 3). These are publicly available databases that contain over 850 genes and proteins and include over 18,000 interactions involved in immune processes and pathways as determined by GO and experimental analyses [21,28]. Of the 62 identified DEG, 29 were identified as immune-related genes: *CCL20*, *PTGS2*, *NR4A3*, *BTG2*, *EGR3*, *EGR1*, *SELE*, *CXCL2*, *C6*, *BDKRB1*, *CSF3*, *MSC*, *ATG10*, *IER3*, *NFKBIZ*, *MAP3K8*, *NFKBIA*, *NFKB1*, *SUSD4*, *VCAM1*, *TNFAIP3*, *LIF*, *F3*, *NFATC2*, *CXCL8*, *CCL2*, *DUSP1*, *CCL8*, *LYZ* (Table 1, bolded text; Table 3).

Functional pathways analyses with GENEWIZ and Advaita iPathwayGuide were performed on the 62 DEG, and common functional pathways were ranked by statistical significance as determined by number of DEG and *p*-adj value (Table 3). Functional pathways identified were TNF signaling, AGE-RAGE signaling in diabetes, IL-17 signaling, NF-κB signaling, chemokine signaling, NOD-like receptor signaling, MAPK signaling, TLR signaling, T cell receptor signaling, complement and coagulation cascades, B cell receptor signaling, RIG-I-like receptor signaling, cytokine-cytokine receptor interaction, Th17 activation, and Th1 and Th2 activation (Table 3). Furthermore, fluid shear stress and atherosclerosis, C-type receptor signaling, osteoclast differentiation, VEGF signaling, cellular senescence, relaxin signaling, oxytocin signaling, seratonergic synapse, neurotrophin signaling, and parathyroid hormone synthesis/secretion/action pathways were also found (Table 3).

Further ontological analyses of RNA sequencing data identified over 100 total pathways and 26 functional pathways affected in NPSC by xenogeneic complement. Functional pathway analyses revealed that the top affected pathways are related to immune regulation and survival, which were categorized into three biological clusters and will be discussed further: innate immune signaling (Figure S2A), cytokine interactions (Figure S2B), and T cell regulation (Figure S2C).

#### 3.5.1. Innate Immune Signaling Pathways

Five different innate immune signaling pathways were identified in functional pathway analyses: the complement and coagulation cascade, C-type lectin receptor (CLR) signaling, nucleotide oligomerization domain (NOD)-like receptor (NLR) signaling, toll-like receptor (TLR) signaling, and retinoic acid-inducible gene I (RIG-I)-like receptor (RLR) signaling (Figure S2A). Since NPSC were exposed to the complement, it is not unexpected to see that some complement and coagulation genes are significant DEG (Figure S3A). This cascade contains four DEG (*C6*, *SUSD4*, *SERPINB2*, *F3*) and two GOI (*C6*, *SUSD4*) (Figure S3A). These genes may be affecting the complement and coagulation cascade through inhibition of complement activation on NPSC, production of terminal complement components, and facilitating coagulation (Figure S3A).

Complement components can also act as molecular sensors that interact with various pattern recognition receptors (PRR) to initiate signaling cascades important in immune cell activation or apoptosis [29]. As follows, the other four affected innate immune signaling pathways are different PRR: CLR (Figure S3B), NLR (Figure S3C), RLR (Figure S3D), and TLR (Figure S3E). CLR are transmembrane receptors that are activated by pathogen-associated carbohydrates to induce phagocytoses of the pathogen and subsequent antigen presentation, making them mainly associated with APC [30,31]. NLR are intracellular PRR that detect pathogen-related factors phagocytosed into the cell to initiate inflammation and/or apoptosis [30,32]. RLR are activated by interaction with intracellular RNA viruses and lead to the production of antiviral factors and interferons [30]. TLR have intracellular and ectodomains that are activated by various microbial factors and initiate a cascade that leads to production of proinflammatory factors such as TNF (to be discussed later) [30,32]. These pathways have three DEG/GOI in common: *CXCL8*, *NFKB1*, and *NFKBIA*, along with the GOI *CCL2* (NLR), *PTGS2* (CLR), and *TNFAIP3* (NLR).

While these PRR are triggered by different types of PAMP, their downstream signaling effects are similar [33]. When bound to their respective trigger, PRR induce the production of inflammatory mediators and aid in activation of leukocytes, particularly innate immune cells such as macrophages and dendritic cells (antigen presenting cells, APC) [33,34]. PRR signaling specifically initiates the response against foreign material such as transplanted cells, and with APC responding against transplanted cells, they can activate T cells to mount an adaptive immune response against the graft. The CLR, NLR, RLR, and TLR pathways are projected to have decreased production of pro-inflammatory factors with increases in survival and regulatory factors such as ERG3, PTGS2, and IL8 (Figure S3B–E).

#### 3.5.2. Cytokine Signaling

Five different pathways involved in cytokine signaling were identified in functional pathway analyses: chemokine signaling, IL-17 signaling, cytokine-cytokine receptor interactions, NF-κB signaling, and tumor necrosis factor (TNF) signaling (Figure S2B). Common DEG/GOI between these pathways are *CCL2*, *CCL20*, *CXCL8*, *NFKB1*, *NFKBIA*, *PTGS2*, *TNFAIP3*, and *VCAM1*. Cytokine signaling pathways are among the pathways that contain the most DEG after NPSC exposure to complement. As cytokines are critical in both shaping and executing immune responses, regulating these pathways may be a mechanism NPSC utilize in survival.

Chemokine signaling pathways are important in the migration of immune cells during immune response, and when chemokine signaling is blocked in transplantation models through small molecule targeting of receptors, survival is increased (Figure S4A) [35,36]. IL-17 is a cytokine that modulates immune activity by activating T cells and neutrophils, and can also induce the NF-κB pathway, lead to expression of inflammatory cytokines and antimicrobial molecules, and aid in leukocyte mobilization (Figure S4B) [37]. IL-17 has been closely linked with the inflammatory effector T cell subtype Th17, which is known to produce inflammatory cytokines and to recruit and activate leukocytes (particularly neutrophils) [38]. Cytokines activate immune cells, initiate the expansion and proliferation of immune cells, and cause cell death of target cells, and these functions are initiated by cytokine–cytokine receptor interactions (Figure S4C) [39]. NF-κB signaling is an integral part of inflammatory immune responses by regulation of effector immune cell survival and activation (Figure S4D) [40]. TNF signaling regulates inflammation and induces caspase-mediated cell death (Figure S4E) [41,42,43]. TNF is considered a proinflammatory cytokine important in effector immune response and is associated with rejecting grafts [44].

#### 3.5.3. T Cell Regulation

Four pathways affected by complement exposure are related to T cell regulation: mitogen-activated protein kinase (MAPK) signaling, T cell receptor (TCR) signaling, Th1/Th2 cell activation, and Th17 activation (Figure S2C). These pathways have four DEG between them including *MAP3K8*, *NFATC2*, *NFKB1*, and *NFKBIA*. T cells are central to adaptive immunity; thus, the regulation of the activation of these cells would be critical in survival of chronic complement exposure and graft rejection. Perturbation analyses of these pathways predicts that NPSC may influence induction and recruitment of T cell subtypes to increase infiltrate of Tregs and to decrease infiltrate of effector T cells (Th1, Th17, and cytotoxic T cells), which are heavily implicated in graft rejection.

MAPK signaling plays a role in T cell selection, activation, and expansion (Figure S5A) [45]. T cell activation starts with engagement of the TCR on the T cell to the major histocompatibility complex (MHC) on an antigen-presenting cell. The TCR initiates a signaling cascade that promotes survival, activation, expansion, and cytokine production, all which ensure an effective adaptive immune response (Figure S5B). T cell activation pathways were also affected. Th1 cells are mediators of proinflammatory immune responses, while Th2 cells are anti-inflammatory and associated with wound healing, thus skewing the Th1/Th2 cell infiltrate ratio toward a stronger Th2 response that would aid in graft survival (Figure S5C). In transplantation, Th2 cells and Th2-related cytokines are associated with surviving graft outcomes and even continue to promote extended survival [46,47,48]. As discussed previously, Th17 cells are also inflammatory cells usually observed at mucosal surfaces, and have been associated with tissue destruction in graft rejection (Figure S5D) [49,50].

## 4. Discussion

Xenotransplantation is now being performed in the clinic, so creating a protective environment to increase the viability of porcine xenotissue is of the utmost importance for patients awaiting transplants. SC are an integral part of creating a similar environment within the testis. To understand how SC modulate the complement system in immune protection, we collected total RNA from NPSC after a 90 min exposure to activated human complement (NHS) and baseline NPSC for RNA sequencing, which identified 62 DEG. qPCR was conducted to validate RNA sequencing results on the top 12 GOI as determined by LFC and association with the top affected pathways from GO analyses. For the first time, we have shown that SC express A20 and secrete CCL2.

The development of graft tolerance is a topic of much interest in the transplantation field. Tolerance is thought to result from factors that promote anti-inflammatory and immune suppressive responses, and indeed many DEG from NPSC complement exposure are associated with immune suppression and accommodation: *CCL2*, *CCL20*, *PTGS2* (COX2), *CXCL8* (IL-8), *NFKBIA* (IκBα), *TNFAIP3* (A20), and *VCAM1*. CCL2 has been shown to activate Tregs, which are associated with surviving grafts [25,26]. CCL20 is a Treg recruiting cytokine that plays roles in both immunological tolerance and autoimmune inflammation [24]. COX2 mediates the trafficking of Tregs into inflammatory environments and in prostaglandin synthesis for membrane repair [51]. IL-8 is an anti-inflammatory cytokine that can produce an immune suppressive environment [52]. Tregs, macrophages, and dendritic cells produce IL-8 to attract neutrophils and myeloid derived suppressor cells (MDSC), which are associated with an immune-suppressive environment, particularly in tumors [53,54,55,56]. IκB prevents NF-κB translocation into the nucleus, inhibiting its ability to transcribe proinflammatory genes [40]. A20 has recently been established as a transplant accommodation marker, even to the point of prolonging islet transplant survival [22]. VCAM-1 is an adhesion molecule that interacts with ICAM-1 on lymphocytes to increase adhesion, delay migration, and decreases activation of effector T cells to lower inflammation [57].

Increased expression of these factors could be an important mechanism in graft protection and are worthy of future study. For this study, we exposed NPSC to complement for 1.5 h, as this was the standard amount of time for NPSC exposure to human complement in our past studies [10,11]. The 62 DEG that were identified are important in defining the initial response to complement. However, it would be interesting to analyze gene expression changes that have had a longer complement exposure (12–24 h). This may reveal additional significant pathways affected by complement, and is the focus of future studies.

Keeping a broad view in mind, analyses of functional pathways within the three biological clusters (innate immune signaling, cytokine signaling, and T cell regulation) revealed a similar pattern of effects that may explain how SC create and maintain an immune protective environment. The DEG involved in the complement and coagulation cascade, CLR, NLR, RLR, and TLR are projected to inhibit apoptosis, decrease inflammation, restrict effector immune cell activation, recruit suppressive immune cells, and repair cell membrane damage within NPSC. The cytokine signaling pathways have many DEG in common, and Advaita pathway analyses project a decrease in caspase activation, reduced expression of inflammatory mediators, and inhibition of Th1 and Th17 cell function [22,58]. Complement, PRR, and cytokine signaling are important in initial pathogen recognition and response, which feed into the eventual activation of APC and T cells [54,59]. Complement-influenced DEG in SC are projected to affect T cell regulation that may aid in recruiting and generating Tregs and anti-inflammatory Th2 cells, inhibiting induction of inflammatory T cells such as Th1/Th17 and cytotoxic T cells (CTL), and establishing an immune-suppressed environment conducive for graft survival. Indeed, in the SC graft environment, we observe increased cell survival, increased infiltrate of Tregs, decreased infiltrate of effector T cells and macrophages, elevated anti-inflammatory cytokines (e.g., IL-10, thrombospondin 1, and galectin-1), and reduced pro-inflammatory cytokines such as tumor necrosis factor alpha (TNFα) and interferon gamma (IFNγ) [26,60,61,62].

**Figure 3:**
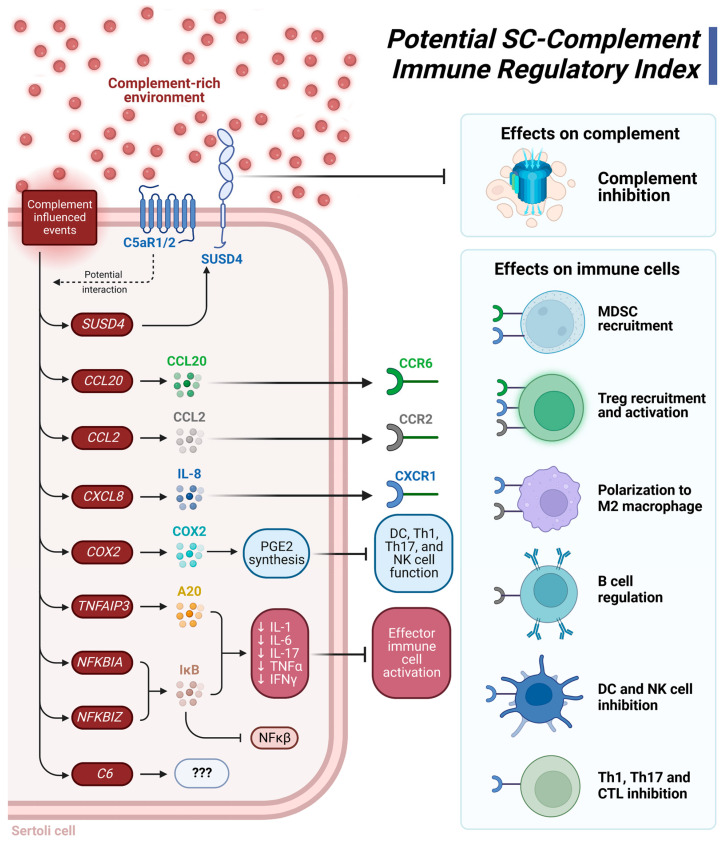
Suggested Sertoli cell (SC)-complement immune regulatory index. A complement-rich environment upregulates expression of the genes *SUSD4*, *CCL20*, *CCL2*, *CXCL8* (IL-8), *COX2* (PTGS2), *TNFAIP3* (A20), *NFKBIA*, *NFKBIZ*, and *C6*. SUSD4 is a complement inhibitor protein that prevents complement-mediated damage. When bound to their respective receptors, the cytokines CCL20 (CCR6 receptor), CCL2 (CCR2 receptor), and CXCL8 (CXCR1 receptor) recruit MDSC and Tregs, polarize macrophages to an anti-inflammatory M2 phenotype, regulate B cell response, and can inhibit responses from dendritic cells, Th1 cells, Th17 cells, and CTL. COX2 is integral to PGE2 synthesis, an immune suppressive molecule that inhibits the function of inflammatory DC, Th1, Th17, and NK cells. The NF-κβ inhibitor, IκB, is encoded by *NFKBIA* and *NFKBIZ*. IκB, along with A20 are graft accommodation markers as they decrease transcription of the genes for the proinflammatory cytokines IL-1, IL-6, IL-17, TNFα, and IFNγ. A decrease in this factor also decreases effector immune cell activation. C6 is also upregulated by complement exposure, but as the only known function of C6 is as part of the MAC, the purpose of its upregulation is a mystery. Figure was created using BioRender.

Considering these DEG and functional pathway analyses, we present a potential SC-complement immune regulatory index (Figure 3). The xenogeneic complement-rich environment influences signaling events that lead to increased expression of *SUSD4*, which would inhibit deleterious complement action; increased expression of genes (i.e., *CCL20*, *CCL2*, *CXCL8*) important in recruiting immune suppressive leukocytes such as Tregs and MDSC; increased expression of other genes (*TNFAIP3*, *NFKBIA*, *NFKBIZ*) that inhibit production of pro-inflammatory cytokines. Elevated levels of *COX2* leads to increased production of the immune suppressive molecule prostaglandin E2 (PGE2) [63] (Figure 3). The range of immune regulation includes complement inhibition, MDSC recruitment, Treg recruitment and activation, M2 macrophage polarization, regulation of B cell responses, inhibition of dendritic cell (DC) and natural killer (NK) cell proinflammatory responses, and inhibition of Th1/Th17 cell and CTL anti-graft action (Figure 3). Altogether, we propose that these effects contribute to graft protection from detrimental immune responses. Future studies should investigate NPSC protein expression changes after longer exposure (12+ h) to NHS. Additionally, functional and mechanistic studies should be conducted in the future to confirm these findings.

## 5. Conclusions

The 62 DEG identified from xenogeneic complement exposure affect immune- and survival-related pathways that have never before been identified as related to the complement system. The pattern of expression for complement components, cytokines, and immunoregulatory markers support the hypothesis that SC generate and maintain an immune modulatory environment conducive to graft survival. SC accomplish this by regulating immune responses in three primary functional means: complement control, expression of immune suppressive cytokines, and activation of immune suppressive T cells and other leukocytes (Figure 3). Each of these functional areas are associated with the establishment of graft tolerance and accommodation. Indeed, SC may be able to affect which leukocytes infiltrate the graft—particularly T cells—to create a tolerogenic environment. The large data sets obtained from this study and these initial analyses of gene expression have great potential to generate future experiments further exploring immune regulation, cell survival, and other factors. The main impact of this study is that we, for the first time, identified potential immunoregulatory pathways affected by complement exposure that may be important in achieving transplant accommodation (Figure 3).

## Figures and Tables

**Table 1:** Significant differentially expressed genes in NPSC after human complement exposure.

Gene Symbol	NPSC Ensembl Number	Human GeneID	LFC	*p*-Adj
***CCL20***	ENSSSCG00000016254	6364	3.613	1.00 × 10^−6^
***PTGS2***	ENSSSCG00000015579	5743	3.529	1.00 × 10^−6^
*KERA*	ENSSSCG00000000917	11081	3.371	1.61 × 10^−4^
***NR4A3***	ENSSSCG00000005385	8013	2.967	1.00 × 10^−6^
*ssc-mir-212* *	ENSSSCG00000019896	N/A	2.416	2.17 × 10^−3^
*ssc-mir-221* *	ENSSSCG00000018698	N/A	2.412	5.41 × 10^−3^
***BTG2*** *	ENSSSCG00000028322	7832	2.278	2.41 × 10^−13^
*F2Z5K9* *	ENSSSCG00000026982	N/A	2.237	5.42 × 10^−3^
***EGR3***	ENSSSCG00000009630	1960	2.048	1.00 × 10^−6^
***EGR1***	ENSSSCG00000014336	1958	2.018	1.00 × 10^−6^
***SELE***	ENSSSCG00000006286	6401	1.986	3.46 × 10^−6^
*SORCS3*	ENSSSCG00000010617	22986	1.869	1.50 × 10^−2^
***CXCL2*** *	ENSSSCG00000008959	2920	1.782	6.32 × 10^−7^
***C6***	ENSSSCG00000016861	729	1.759	3.20 × 10^−2^
***BDKRB1*** *	ENSSSCG00000002501	623	1.666	1.30 × 10^−3^
***CSF3*** *	ENSSSCG00000017482	2438	1.664	2.43 × 10^−2^
*FOXR1*	ENSSSCG00000015101	283150	1.599	9.00 × 10^−3^
***MSC***	ENSSSCG00000006187	9242	1.548	1.00 × 10^−6^
***ATG10*** *	ENSSSCG00000029605	83734	1.538	6.25 × 10^−11^
***IER3***	ENSSSCG00000027607	8870	1.499	1.62 × 10^−5^
*LMCD1*	ENSSSCG00000011538	29995	1.498	2.02 × 10^−6^
*JUNB*	ENSSSCG00000013735	3726	1.424	1.00 × 10^−6^
*MYOC*	ENSSSCG00000015481	4653	1.405	4.68 × 10^−2^
*BHLHE40* *	ENSSSCG00000011534	8553	1.399	5.44 × 10^−4^
***NFKBIZ***	ENSSSCG00000011951	64332	1.355	4.00 × 10^−3^
***MAP3K8***	ENSSSCG00000020705	1326	1.334	2.18 × 10^−4^
*AK7*	ENSSSCG00000002504	122481	1.328	3.80 × 10^−2^
*TGM7*	ENSSSCG00000004712	116179	1.325	4.00 × 10^−2^
***NFKBIA***	ENSSSCG00000001952	4792	1.323	1.00 × 10^−3^
*GEM*	ENSSSCG00000006105	2669	1.293	2.00 × 10^−3^
*GPC3*	ENSSSCG00000012680	2719	1.276	2.10 × 10^−2^
***NFKB1***	ENSSSCG00000030957	4790	1.271	2.40 × 10^−2^
***SUSD4***	ENSSSCG00000011176	55061	1.265	2.20 × 10^−2^
*ADAM7*	ENSSSCG00000009646	8756	1.251	1.20 × 10^−2^
*VEGFA*	ENSSSCG00000001695	7422	1.249	1.62 × 10^−5^
***VCAM1***	ENSSSCG00000006862	7412	1.240	2.00 × 10^−3^
*SHAS2*	ENSSSCG00000005992	3037	1.215	8.00 × 10^−3^
*SNARK* *	ENSSSCG00000027243	81788	1.206	2.17 × 10^−3^
*NR4A2*	ENSSSCG00000015871	4929	1.206	1.00 × 10^−3^
*DUSP10*	ENSSSCG00000010831	11221	1.200	1.69 × 10^−4^
*LOC110259249* *	ENSSSCG00000052816	N/A	1.152	4.00 × 10^−3^
***TNFAIP3***	ENSSSCG00000004154	7128	1.120	1.80 × 10^−2^
*SLCO5A1*	ENSSSCG00000006196	81796	1.109	8.00 × 10^−3^
***LIF*** *	ENSSSCG00000009996	3976	1.105	1.26 × 10^−3^
*KDM6B*	ENSSSCG00000017962	23135	1.100	8.00 × 10^−3^
*SERPINB2*	ENSSSCG00000004890	5055	1.088	3.70 × 10^−2^
***F3***	ENSSSCG00000022447	2152	1.053	5.00 × 10^−3^
*GADD45G* *	ENSSSCG00000009585	N/A	1.048	8.80 × 10^−3^
*NFATC2*	ENSSSCG00000007477	4773	1.027	4.00 × 10^−2^
*PER1* **	ENSSSCG00000017983	5187	0.979	8.00 × 10^−3^
***CXCL8*** **	ENSSSCG00000008953	3576	0.97	4.60 × 10^−2^
***CCL2*** **	ENSSSCG00000017723	6347	0.946	2.00 × 10^−3^
***DUSP1*** **	ENSSSCG00000016991	1843	0.856	3.70 × 10^−2^
*MAP3K21* **	ENSSSCG00000010164	84451	−0.724	4.70 × 10^−2^
*PSAP* **	ENSSSCG00000010281	5660	−0.795	4.80 × 10^−2^
*GATA6* **	ENSSSCG00000003702	2627	−0.805	4.00 × 10^−2^
*TMEM245* **	ENSSSCG00000005444	23731	−0.861	2.20 × 10^−2^
*OXTR* **	ENSSSCG00000021585	5021	−0.864	2.80 × 10^−2^
*IER5L*	ENSSSCG00000005673	389792	−1.272	3.46 × 10^−6^
*CYP1A1*	ENSSSCG00000001906	1543	−1.394	4.50 × 10^−5^
*CCL8* **	ENSSSCG00000017721	N6355A	−2.257	0.026123
***LYZ***	ENSSSCG00000000492	4069	−2.312	3.46 × 10^−6^

* indicates DEG was only identified by GeneWiz. ** indicates DEG was only identified by Advaita. Significant DEG are defined as having a LFC > 0.7 or LFC < −0.7 and *p*-adj < 0.05. Bold text are immune-related genes as identified by IKB [20] and InnateDB [21].

**Table 2:** Immune-related DEG *.

Gene	Name	Immune Function
***ATG10***	Autophagy-related 10	Autophagosome formation
***BDKRB1***	Bradykinin receptor B1	Induces inflammatory responses
***BTG2***	B cell translocation gene 2	Antiproliferative functions
***C6*** **	Complement C6	Formation of MAC
***CCL2*** **	C-C motif ligand 2	Induces monocyte trafficking
***CCL20*** **	C-C motif ligand 20	Antimicrobial cytokine
***CCL8***	C-C motif ligand 8	Antimicrobial cytokine that recruits leukocytes to inflammatory site
***CSF3***	Colony stimulating factor 3	Regulates granulocyte production, function, and differentiation
***CXCL2***	C-X-C motif ligand 2	Recruitment of neutrophils
***CXCL8*** **	C-X-C motif ligand 8	Angiogenesis regulation, infiltration of leukocytes, cell motility and survival
***DUSP1***	Dual specificity phosphatase 1	Decreases TLR signaling and inhibits MAPK induction of IL-6 and IL-8
***EGR1*** **	Early growth response 1	Cell differentiation and mitogenesis
***EGR3***	Early growth response 3	Lymphocyte development
***F3*** **	Coagulation factor III (thromboplastin)	Initiates blood coagulation cascades
***IER3***	Immediate early response 3	Protects against Fas or TNF-α apoptosis
***LIF***	Leukemia inhibitory factor	Differentiation of myeloid cells, immune tolerance
***LYZ*** **	Lysozyme	Antimicrobial, cleavage of peptidoglycans in bacterial cell walls
***MAP3K8***	Mitogen-activated protein kinase kinase kinase 8	Regulates IL-1β and TNF production in macrophages and dendritic cells
***MSC***	Musculin	B cell receptor signaling target
***NFATC2***	Nuclear factor of activated T cells, cytoplasmic, calcineurin-dependent 2	Activates IL-2 transcription through interaction with JUN in T cells
***NFKB1*** **	Nuclear factor of kappa light polypeptide gene enhancer in B cells 1	Regulation of immune and inflammatory response genes; regulation of cell growth
***NFKBIA*** **	Nuclear factor of kappa light polypeptide gene enhancer in B cells inhibitor alpha	Inhibition of NFκB
***NFKBIZ*** **	Nuclear factor of kappa light polypeptide gene enhancer in B cells inhibitor zeta	Inhibits NFκB1 binding to DNA; regulates IL-6 production, regulates TLR and NOD-like receptor inflammation
***NR4A3***	Nuclear receptor subfamily 4, A3	Induces repressive effects in dendritic cells
***PTGS2*** **	Prostaglandin-endoperoxidase synthase 2	Prostaglandin biosynthesis important in inflammation and mitogenesis
***SELE***	Selectin E	Accumulates leukocytes to inflammatory cites; allows for adhesion of leukocytes to endothelium
***SUSD4***	Sushi domain containing 4	Membrane-bound inhibition of alternative and classical complement activation
***TNFAIP3*** **	Tumor necrosis factor, alpha-induced protein 3 (A20)	Restricts TLR, NOD, and RIG-I signaling; negatively regulates NF-κB signaling pathway
***VCAM1*** **	Vascular cell adhesion molecule 1	Mediates leukocyte adhesion to endothelium

* Data retrieved from NCBI Gene Database [27]. ** genes discussed in depth in the text.

**Table 3:** Functional pathways from Advaita iPathwayGuide analyses of DEG.

Pathway Name	#DEG/TG	*p*-Value	Genes
TNF signaling *	10/77	0.00001	***↑CCL20↑PTGS2*** *↑SELE ↑JUNB ↑MAP3K8****↑NFKBIA↑NFKB1****↑VCAM1****↑TNFAIP3↑CCL2***
AGE-RAGE signaling in diabetes	8/73	0.000001	***↑EGR1*** *↑SELE****↑NFKB1*** *↑VEGFA ↑VCAM1 ↑F3****↑CXCL8↑CCL2***
IL-17 signaling *	7/55	0.00001	***↑*** ***CCL20*** ***↑*** ***PTGS2*** ***↑*** ***NFKBIA*** ***↑*** ***NFKB1*** ***↑*** ***TNFAIP3*** ***↑*** ***CXCL8*** ***↑*** ***CCL2***
NF-κB signaling *	6/69	0.000003	***↑PTGS2↑NFKBIA↑NFKB1*** *↑VCAM1****↑TNFAIP3↑CXCL8***
Fluid shear stress and atherosclerosis	6/90	0.000005	*↑* *SELE* ***↑*** ***NFKB1*** *↑* *VEGFA* *↑* *VCAM1* ***↑*** ***CCL2*** *↑* *DUSP1*
C-type lectin receptor signaling *	5/68	0.00002	***↑PTGS2*** *↑EGR3****↑NFKBIA↑NFKB1*** *↑NFATC2*
Chemokine signaling *	5/102	0.00005	***↑*** ***CCL20*** ***↑*** ***NFKBIA*** ***↑*** ***NFKB1*** ***↑*** ***CXCL8*** ***↑*** ***CCL2***
NOD-like receptor signaling *	5/90	0.0003	***↑*** ***NFKBIA*** ***↑*** ***NFKB1*** ***↑*** ***TNFAIP3*** ***↑*** ***CXCL8*** ***↑*** ***CCL2***
MAPK signaling pathway *	5/196	0.01	*↑MAP3KIB****↑NFKB1*** *↑VEGFA ↑DUSP10 ↑DUSP1*
Pathways in cancer	5/339	0.014	***↑PTGS2↑NFKBIA↑NFKB1*** *↑VEGFA****↑CXCL8***
TLR signaling *	4/57	0.0005	*↑* *MAP3KIB* ***↑*** ***NFKBIA*** ***↑*** ***NFKB1*** ***↑*** ***CXCL8***
Osteoclast differentiation	4/72	0.001	*↑JUNB****↑NFKBIA↑NFKB1*** *↑NFATC2*
T cell receptor signaling *	4/67	0.001	*↑MAP3K8****↑NFKBIA↑NFKB1*** *↑NFATC2*
Complement and coagulation cascades *	4/47	0.007	***↑C6*** *↑SERPINB2 ↑F3****↑SUSD4***
B cell receptor signaling	3/46	0.002	***↑NFKBIA↑NFKB1*** *↑NFATC2*
RIG-I-like receptor signaling *	3/38	0.002	***↑*** ***NFKBIA*** ***↑*** ***NFKB1*** ***↑*** ***CXCL8***
Cytokine-cytokine receptor interaction *	3/125	0.002	***↑*** ***CCL20*** ***↑*** ***CXCL8*** ***↑*** ***CCL2***
VEGF signaling	3/38	0.004	***↑PTGS2*** *↑VEGFA ↑NFATC2*
Cellular senescence	3/98	0.005	***↑NFKB1*** *↑NFATC2****↑CXCL8***
Th17 cell differentiation *	3/72	0.006	***↑NFKBIA↑NFKB1*** *↑NFATC2*
Th1 and Th2 cell differentiation *	3/59	0.007	***↑NFKBIA↑NFKB1*** *↑NFATC2*
Relaxin signaling	3/87	0.014	***↑NFKBIA↑NFKB1*** *↑VEGFA*
Oxytocin signaling	3/101	0.038	***↑PTGS2*** *↑NFATC2 ↓OXTR*
Seratonergic synapse	2/61	0.015	***↑PTGS2*** *↑DUSP1*
Neurotrophin signaling	2/80	0.031	***↑*** ***NFKBIA*** ***↑*** ***NFKB1***
Parathyroid hormone synthesis, secretion, and action	2/78	0.035	***↑EGR1*** *↑NR4A2*

* functional pathways discussed in depth in the text. TG: total genes. Genes discussed in depth in the text are in bold. ↑ indicates increased gene expression. ↓ indicates decreased gene expression.

## Data Availability

The data presented in this study are openly available in the NCBI GEO database, accession number GSE221711.

## Supplementary Materials

The following supporting information can be downloaded at: https://www.mdpi.com/article/10.3390/biomedicines11061650/s1, Figure S1: General DEG analyses; Figure S2: Venn diagram of the three NPSC biological clusters affected by complement; Figure S3: Innate immune signaling pathways; Figure S4: Cytokine signaling pathways; Figure S5: T cell regulation; Table S1: Genes of interest PCR primers; Table S2: qPCR quantification of GOI between baseline and complement NSPSC; Table S3: Genes of interest.
