# Supplementary material for: Sertoli Cells Express Accommodation, Survival, and Immunoregulatory Factors When Exposed to Normal Human Serum
Source: Biomedicines. 2023 Jun 6;11(6):1650. doi: 10.3390/biomedicines11061650 (PMC10296330; doi:10.3390/biomedicines11061650)
Supplement: Supplementary file 1 [file biomedicines-11-01650-s001.zip › biomedicines-2422068-Figure S1.pdf]

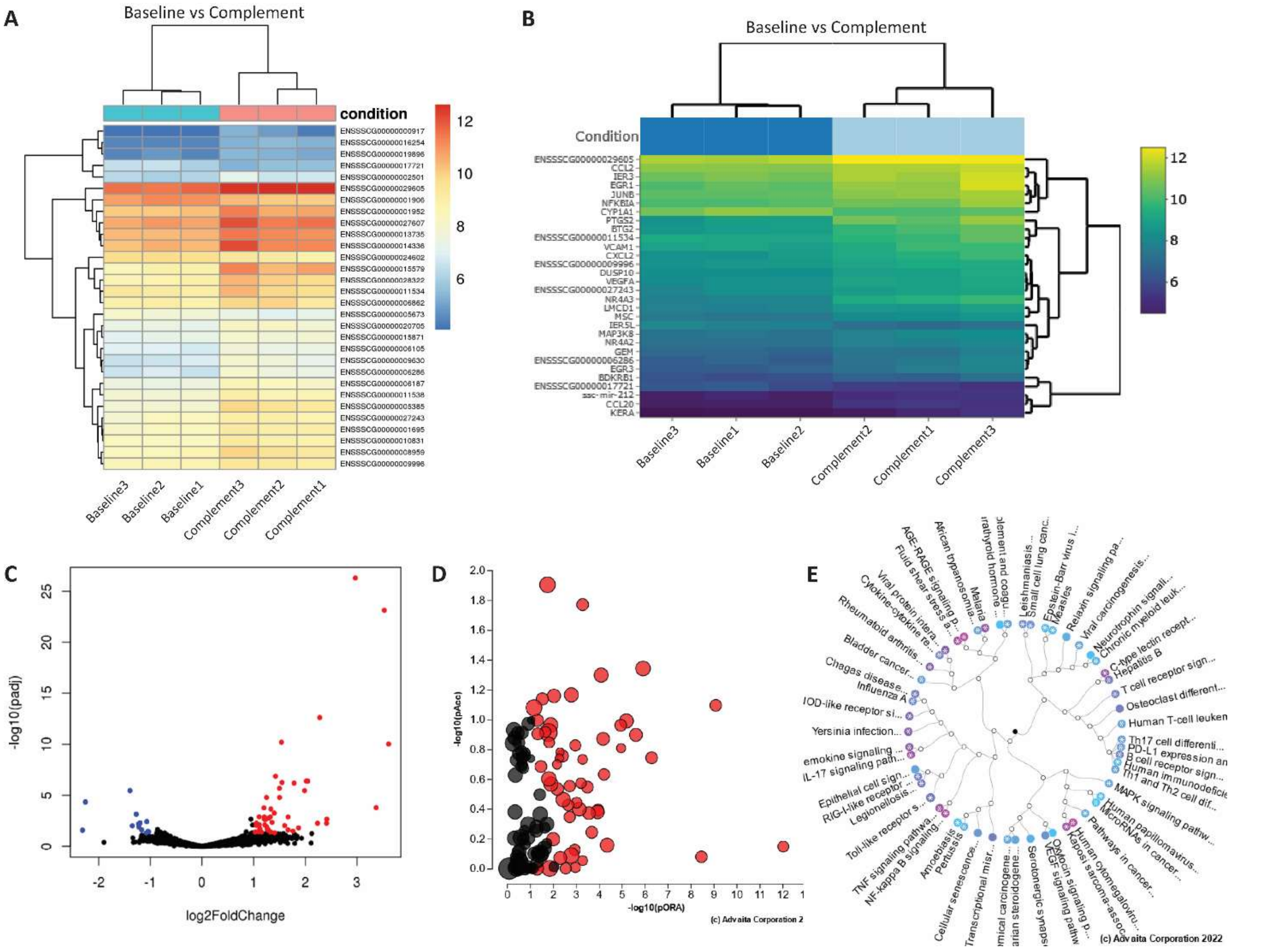

Figure S1. General DEG analyses. (A) Biclustering of top significant adjusted p-values allows for the visualization of clusters of genes (rows) and conditions (columns) in an expression matrix. (B) Heatmap of top 30 differentially expressed genes to visualize the top 30 significant differentially expressed genes. Scale indicates sorting by p-values, which is plotted by log2 transformed expression valleys to identify co-regulated genes across conditions. (C) Volcano plot visualization of DEG showing the global transcriptional change across baseline and complement NPSC groups. Each point in the scatter plot represents a gene. LFC of each gene is represented on the x-axis and the log10 of its adjusted p-value is on the y-axis. Genes with p-adj < 0.05 and LFC > 0.7 are up-regulated genes indicated by red dots (53 genes). Genes with p-adj < 0.05 and a LFC < -0.7 are down-regulated genes indicated by blue dots (9 genes). (D) Pathway plot of functional pathways (black or red dots) affected by complement exposure were plotted by -log10(pAcc) versus -log10(pORA) to visualize impact analysis. Impact analysis takes into account DEG enrichment in a pathway (over-representation) and pathway topology. The x-axis is the p-value of classical over-representative analysis (pORA) and the y-axis is the p-value of total perturbation accumulation in a pathway (pACC). Dot size indicates the number of DEG in the pathway and color indicates significance with red being significant (p<0.01) and black not significant (p>0.01). (E) Dendrogram of pathway-DEG relationships, which is a cluster analyses that allows for visualization of relationships between GO terms and the genes they have in common. The more genes in common, the closer the GO terms are to each other. Leaf nodes (GO term) with at least one DEG in common are indicated by a parent node. Pink indicates more DEG and blue indicates less DEG. White asterisks denote GO terms involved in immunity and infection. Figures created by Advaita.
