# Supplementary material for: Sertoli Cells Express Accommodation, Survival, and Immunoregulatory Factors When Exposed to Normal Human Serum
Source: Biomedicines. 2023 Jun 6;11(6):1650. doi: 10.3390/biomedicines11061650 (PMC10296330; doi:10.3390/biomedicines11061650)
Supplement: Supplementary file 1 [file biomedicines-11-01650-s001.zip › biomedicines-2422068-Figure S2.pdf]

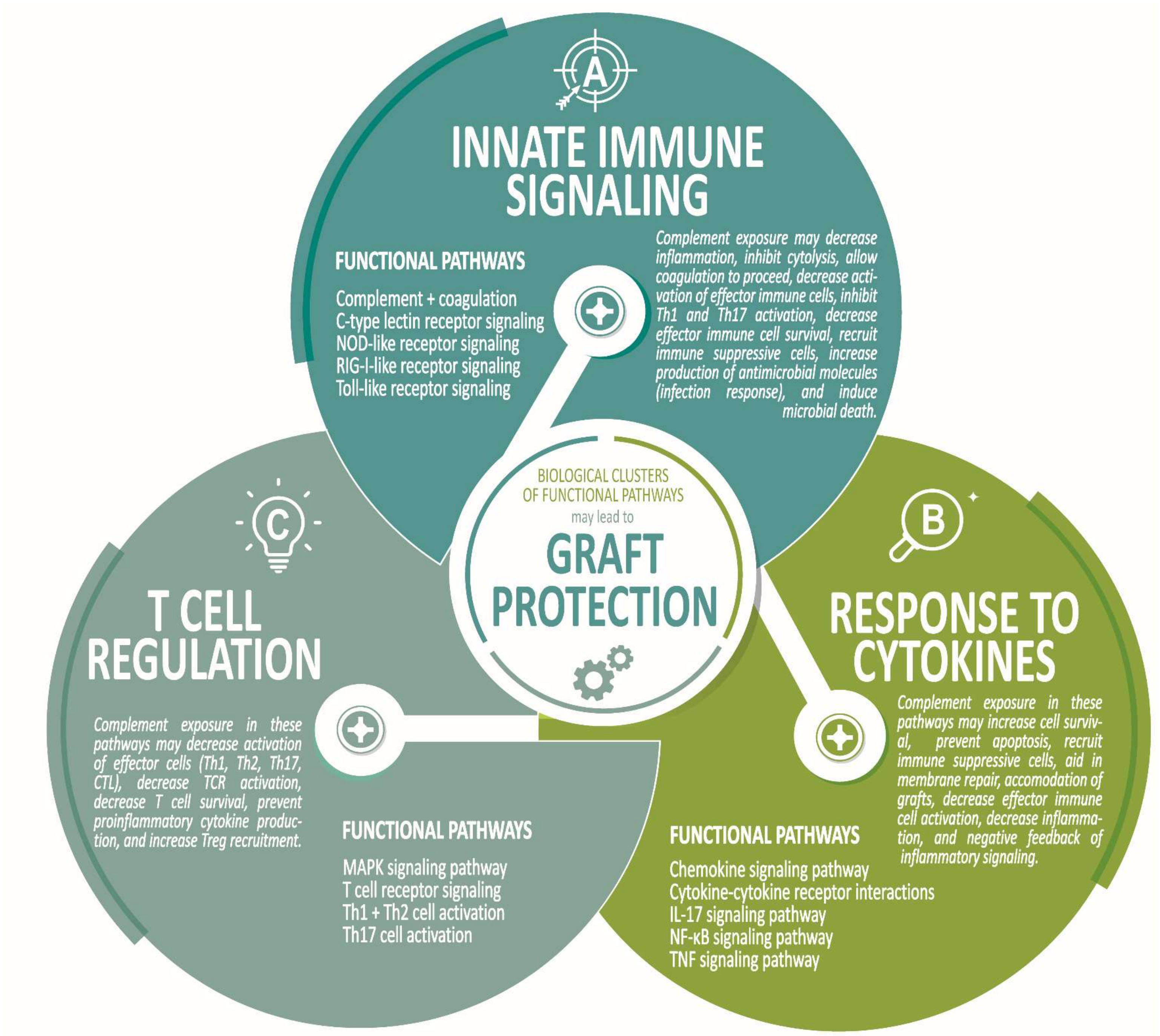

Figure S2. Venn diagram of the three NPSC biological clusters affected by complement. NPSC functional pathways are divided into three biological clusters: innate immune signaling (A), response to cytokines (B), and T cell regulation (C). Each circle contains a list of the functional pathways contained therein and a description of the possible effects complement may be having on these pathways.
