# Supplementary material for: Sertoli Cells Express Accommodation, Survival, and Immunoregulatory Factors When Exposed to Normal Human Serum
Source: Biomedicines. 2023 Jun 6;11(6):1650. doi: 10.3390/biomedicines11061650 (PMC10296330; doi:10.3390/biomedicines11061650)
Supplement: Supplementary file 1 [file biomedicines-11-01650-s001.zip › biomedicines-2422068-Figure S3.pdf]

A

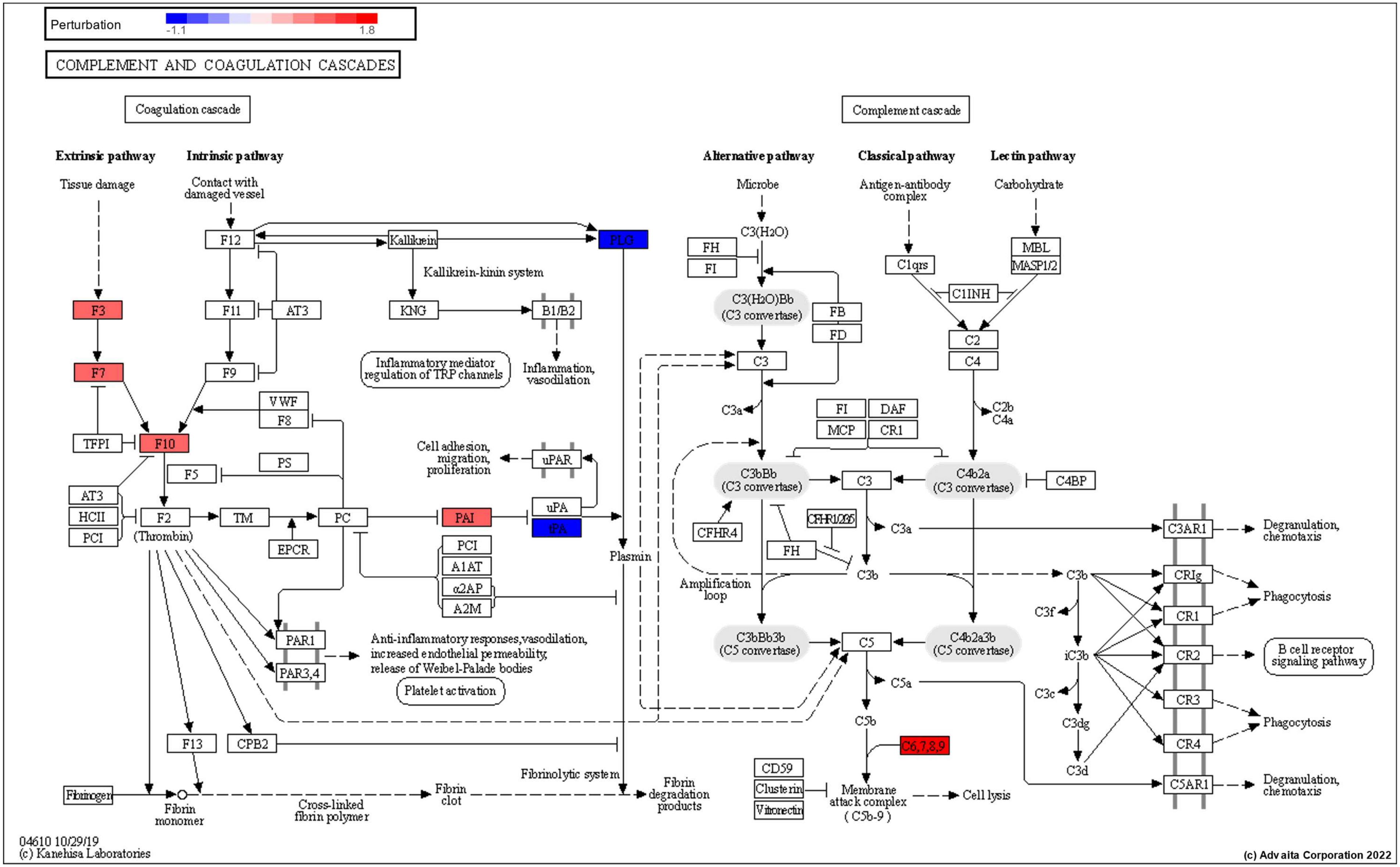
Perturbation
-1.1
1.8
COMPLEMENT AND COAGULATION CASCADES
Coagulation cascade
Complement cascade
Extrinsic pathway
Intrinsic pathway
Alternative pathway
Classical pathway
Lectin pathway
Tissue damage
Contact with damaged vessel
Microbe
Antigen-antibody complex
Carbohydrate
F12
Kallikrein
PLG
F3
F11
AT3
KNG
B1/B2
Kallikrein-kinin system
F7
F9
Inflammatory mediator regulation of TRP channels
Inflammation, vasodilation
VWF
F8
TFPI
F10
PS
Cell adhesion, migration, proliferation
uPAR
F5
AT3
HCII
PCI
F2
(Thrombin)
TM
PC
PAI
uPA
tPA
EPCR
PCI
A1AT
α2AP
A2M
Plasmin
PAR1
PAR3,4
Anti-inflammatory responses,vasodilation, increased endothelial permeability, release of Weibel-Palade bodies
Platelet activation
F13
CPB2
Fibrinogen
Fibrin monomer
Cross-linked fibrin polymer
Fibrin clot
Fibrinolytic system
Fibrin degradation products
FH
FI
C3(H2O)
C3(H2O)Bb (C3 convertase)
FB
FD
C3
C3a
C1qrs
MBL
MASP1/2
C1INH
C2
C4
C2b
C4a
FI
DAF
MCP
CR1
C3bBb (C3 convertase)
C3
C4b2a (C3 convertase)
C4BP
CFHR4
CFHR1/2/3/5
FH
C3a
C3b
Amplification loop
C3AR1
Degranulation, chemotaxis
C3b
CRIg
Phagocytosis
CR1
C3f
iC3b
C3bBb3b (C5 convertase)
C5
C4b2a3b (C5 convertase)
CR2
B cell receptor signaling pathway
C5a
C3c
CR3
C5b
C3dg
Phagocytosis
CR4
C6,7,8,9
C3d
CD59
Clusterin
Vitronectin
Membrane attack complex (C5b-9)
Cell lysis
C5AR1
Degranulation, chemotaxis
04610 10/29/19
(c) Kanehisa Laboratories
(c) Advaita Corporation 2022

B

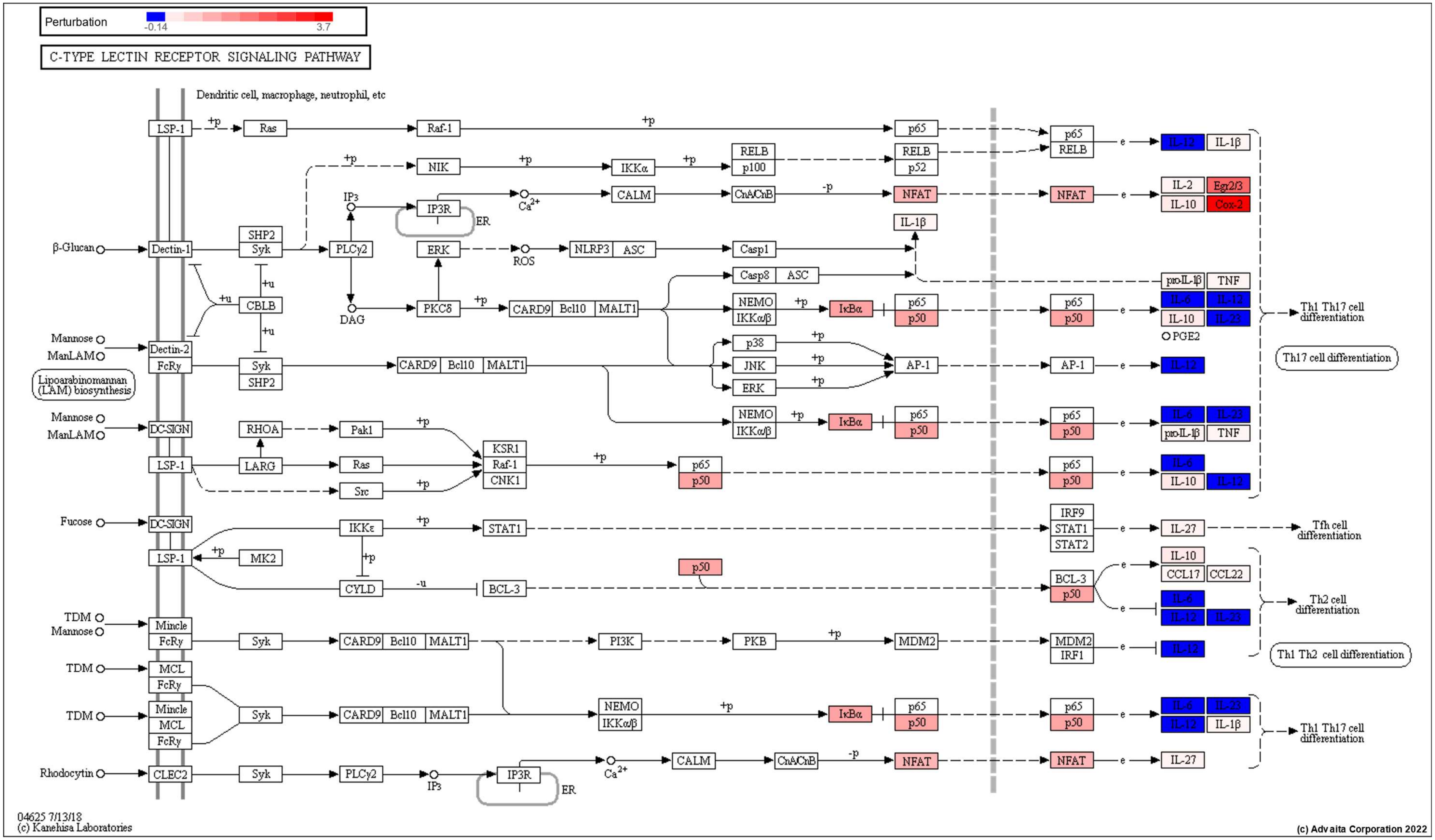

Perturbation
-0.14
3.7
C-TYPE LECTIN RECEPTOR SIGNALING PATHWAY
Dendritic cell, macrophage, neutrophil, etc
LSP-1
+p
Ras
Raf-1
+p
p65
p65
RELB
e
IL-12
IL-1β
NIK
IKKα
RELB
p100
RELB
p52
IP3
IP3R
ER
Ca2+
CALM
CnA/CnB
-p
NFAT
NFAT
IL-2
Egr2/3
IL-10
Cox-2
IL-1β
β-Glucan
Dectin-1
SHP2
Syk
PLCγ2
ERK
ROS
NLRP3
ASC
Casp1
+u
CBLB
Casp8
ASC
pro-IL-1β
TNF
DAG
PKCδ
CARD9
Bcl10
MALT1
NEMO
IKKα/β
IκBα
p65
p50
IL-6
IL-12
IL-10
IL-23
PGE2
Th1 Th17 cell differentiation
Mannose
ManLAM
Dectin-2
FcRγ
Syk
SHP2
p38
JNK
ERK
AP-1
AP-1
Th17 cell differentiation
Lipoarabinomannan (LAM) biosynthesis
DC-SIGN
RHOA
Pak1
KSR1
Raf-1
CNK1
LARG
Ras
Src
Fucose
IKKε
STAT1
IRF9
STAT1
STAT2
IL-27
Tfh cell differentiation
MK2
CYLD
-u
BCL-3
BCL-3
CCL17
CCL22
Th2 cell differentiation
TDM
Mincle
PI3K
PKB
MDM2
MDM2
IRF1
Th1 Th2 cell differentiation
MCL
Rhodocytin
CLEC2
04625 7/13/18
(c) Kanehisa Laboratories
(c) Advaita Corporation 2022

C

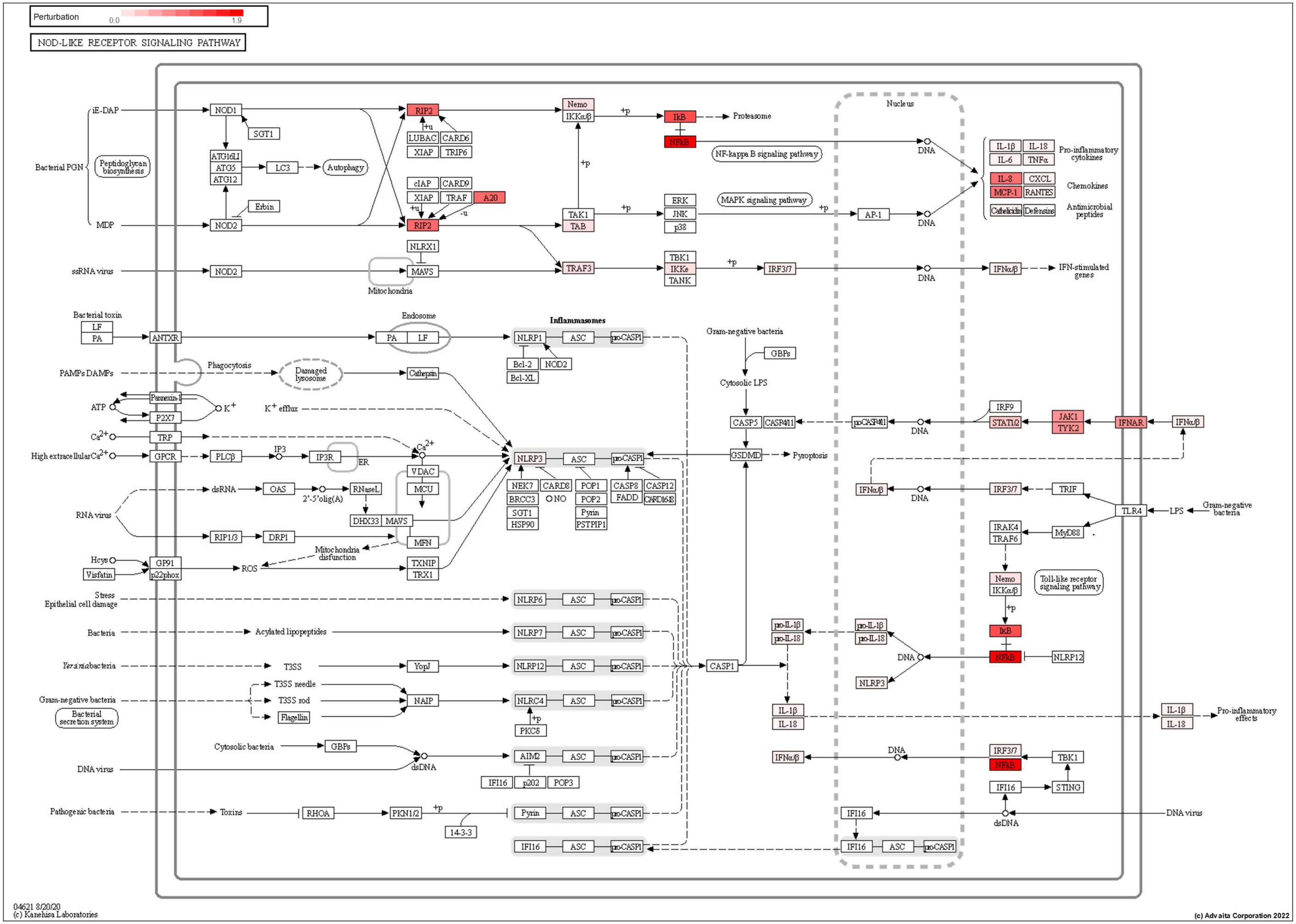
Perturbation
0.0
1.9
NOD-LIKE RECEPTOR SIGNALING PATHWAY
iE-DAP
NOD1
SGT1
RIP2
+u
LUBAC
CARD6
XIAP
TRIP6
Nemo
IKKα/β
+p
IkB
Proteasome
NFkB
NF-kappa B signaling pathway
Nucleus
DNA
IL-1β
IL-18
Pro-inflammatory cytokines
IL-6
TNFα
IL-8
CXCL
Chemokines
MCP-1
RANTES
Cathelicidin
Defensins
Antimicrobial peptides
Bacterial PGN
Peptidoglycan biosynthesis
ATG16L1
ATG5
ATG12
LC3
Autophagy
Erbin
cIAP
CARD9
XIAP
TRAF
A20
+u
-u
MDP
NOD2
RIP2
TAK1
TAB
+p
ERK
JNK
p38
MAPK signaling pathway
AP-1
NLRX1
ssRNA virus
NOD2
MAVS
Mitochondria
TRAF3
TBK1
IKKe
TANK
IRF3/7
IFNα/β
IFN-stimulated genes
Bacterial toxin
LF
PA
ANTXR
Endosome
PA
LF
Inflammasomes
NLRP1
ASC
proCASP1
Bcl-2
NOD2
Bcl-XL
Gram-negative bacteria
GBPs
Cytosolic LPS
PAMPs DAMPs
Phagocytosis
Damaged lysosome
Cathepsin
ATP
Pannexin-1
K+
K+ efflux
P2X7
IRF9
STAT1/2
JAK1
TYK2
IFNAR
IFNα/β
CASP5
CASP4/11
proCASP4/11
Ca2+
TRP
Ca2+
High extracellularCa2+
GPCR
PLCβ
IP3
IP3R
ER
NLRP3
ASC
proCASP1
GSDMD
Pyroptosis
VDAC
MCU
NEK7
CARD8
POP1
CASP8
CASP12
BRCC3
NO
POP2
FADD
CARD16/18
SGT1
Pyrin
HSP90
PSTPIP1
dsRNA
OAS
2'-5'olig(A)
RNaseL
RNA virus
DHX33
MAVS
IFNα/β
IRF3/7
TRIF
TLR4
LPS
Gram-negative bacteria
IRAK4
TRAF6
MyD88
RIP1/3
DRP1
MFN
Mitochondria disfunction
Hcys
GP91
p22phox
Visfatin
ROS
TXNIP
TRX1
Nemo
IKKα/β
Toll-like receptor signaling pathway
Stress
Epithelial cell damage
NLRP6
ASC
proCASP1
Bacteria
Acylated lipopeptides
NLRP7
pro-IL-1β
pro-IL-18
pro-IL-1β
pro-IL-18
IkB
Yersinia bacteria
T3SS
YopJ
NLRP12
CASP1
DNA
NFkB
NLRP12
T3SS needle
NLRP3
Gram-negative bacteria
T3SS rod
NAIP
NLRC4
Bacterial secretion system
Flagellin
PKCδ
IL-1β
IL-18
IL-1β
IL-18
Pro-inflammatory effects
Cytosolic bacteria
GBPs
dsDNA
AIM2
DNA virus
IFI16
p202
POP3
IFNα/β
DNA
IRF3/7
NFkB
TBK1
IFI16
STING
Pathogenic bacteria
Toxins
RHOA
PKN1/2
+p
Pyrin
14-3-3
IFI16
DNA virus
dsDNA
IFI16
ASC
proCASP1
04621 8/20/20
(c) Kanehisa Laboratories
(c) Advaita Corporation 2022

D

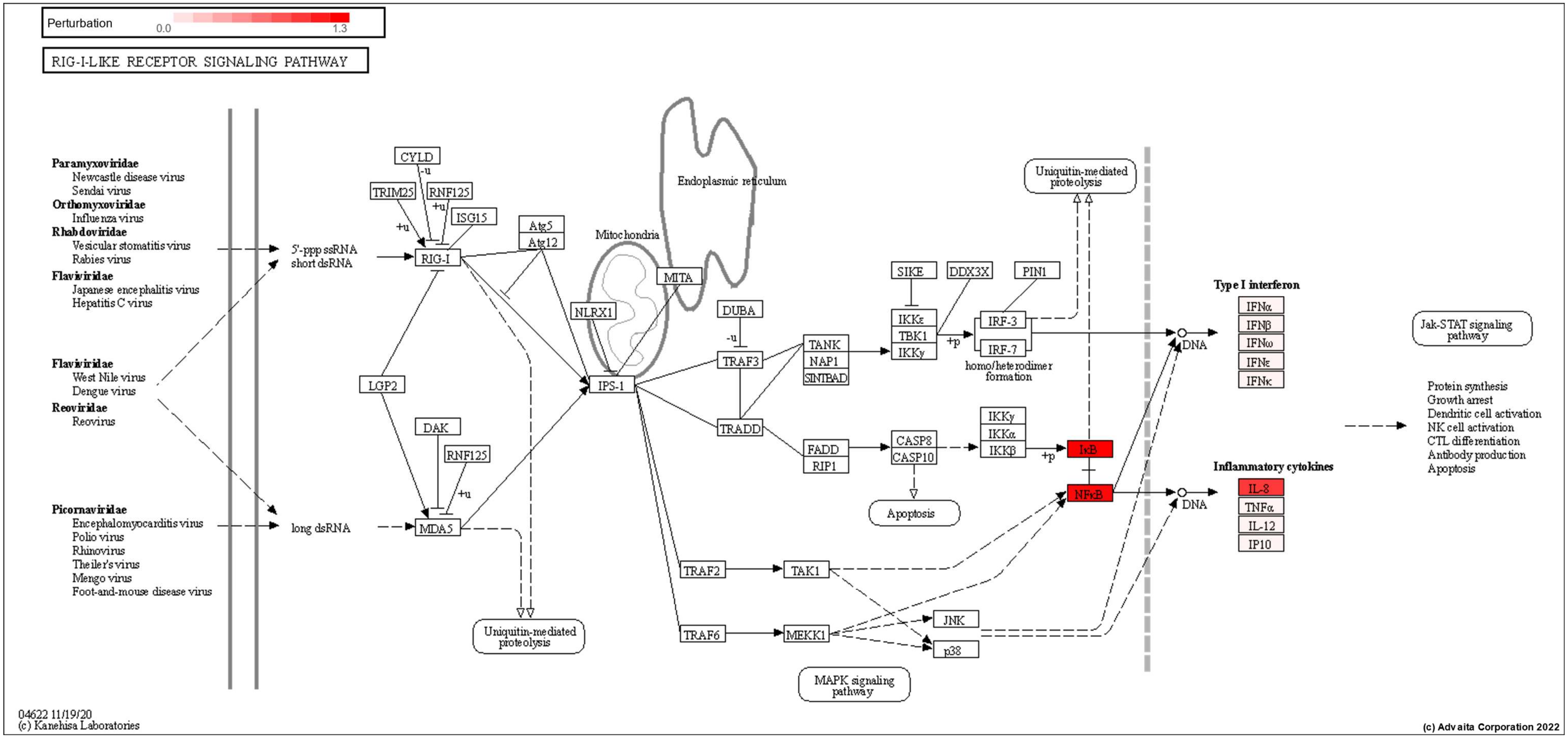
Perturbation
0.0
1.3
RIG-I-LIKE RECEPTOR SIGNALING PATHWAY
Paramyxoviridae
Newcastle disease virus
Sendai virus
Orthomyxoviridae
Influenza virus
Rhabdoviridae
Vesicular stomatitis virus
Rabies virus
Flaviviridae
Japanese encephalitis virus
Hepatitis C virus
Flaviviridae
West Nile virus
Dengue virus
Reoviridae
Reovirus
Picornaviridae
Encephalomyocarditis virus
Polio virus
Rhinovirus
Theiler's virus
Mengo virus
Foot-and-mouse disease virus
5'-ppp ssRNA
short dsRNA
long dsRNA
CYLD
TRIM25
RNF125
ISG15
RIG-I
Atg5
Atg12
LGP2
DAK
RNF125
MDA5
Uniquitin-mediated proteolysis
Mitochondria
Endoplasmic reticulum
NLRX1
MITA
IPS-1
DUBA
TRAF3
TANK
NAP1
SINTBAD
TRADD
FADD
RIP1
CASP8
CASP10
Apoptosis
SIKE
DDX3X
PIN1
IKKε
TBK1
IKKγ
IRF-3
IRF-7
homo/heterodimer formation
IKKγ
IKKα
IKKβ
IκB
NFκB
Uniquitin-mediated proteolysis
TRAF2
TAK1
TRAF6
MEKK1
JNK
p38
MAPK signaling pathway
DNA
DNA
Type I interferon
IFNα
IFNβ
IFNω
IFNε
IFNκ
Inflammatory cytokines
IL-8
TNFα
IL-12
IP10
Jak-STAT signaling pathway
Protein synthesis
Growth arrest
Dendritic cell activation
NK cell activation
CTL differentiation
Antibody production
Apoptosis
04622 11/19/20
(c) Kanehisa Laboratories
(c) Advaita Corporation 2022

E

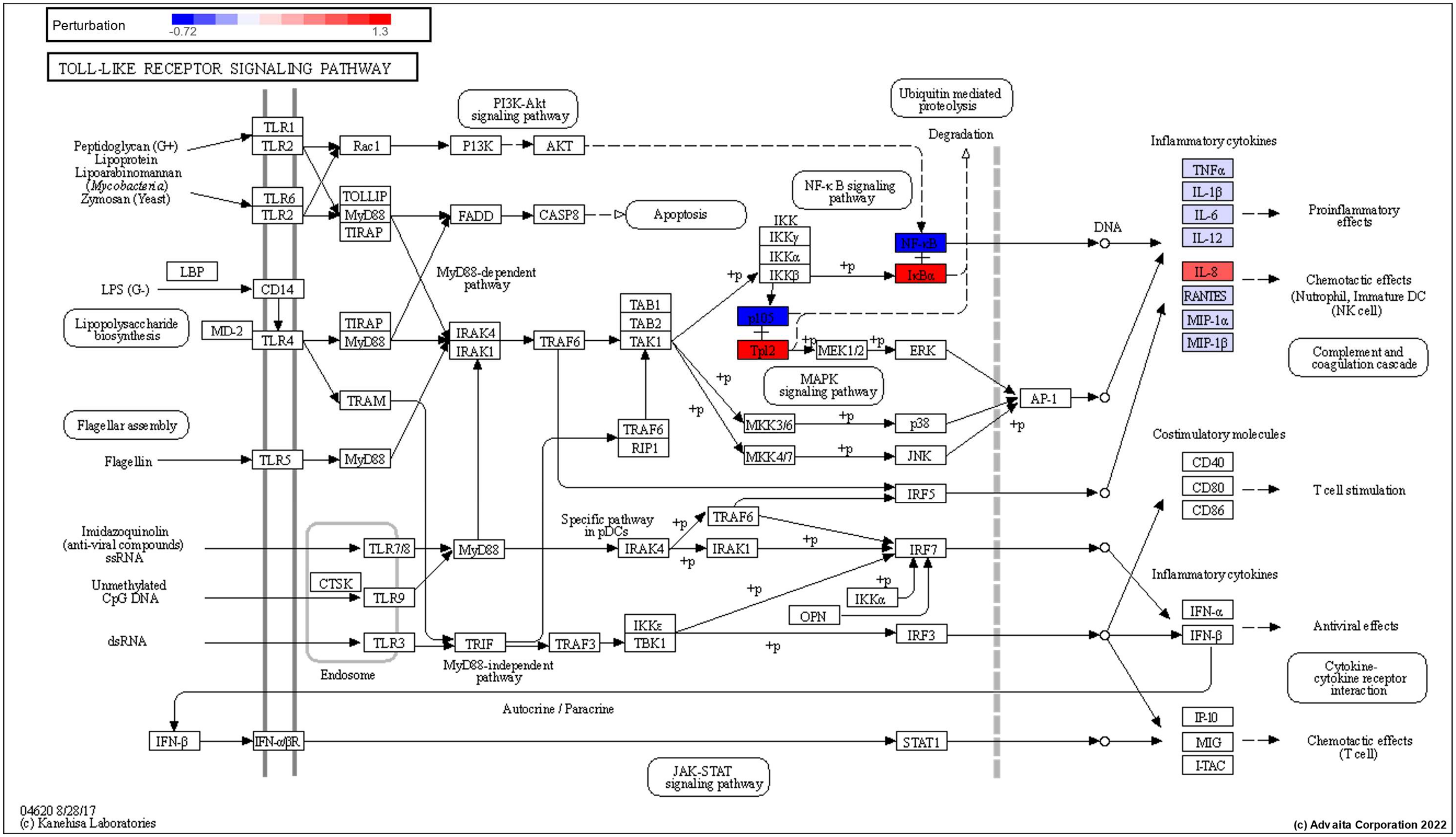

Figure S3. Innate immune signaling pathways. Innate immune pathways in NPSC affected by NHS include (A) the complement and coagulation cascade, (B) CLR signaling, (C) NLR signaling, (D) RLR signaling, and (E) TLR signaling pathways. Pathway perturbations are represented. Red highlighted genes are projected to be significantly upregulated while blue highlighted genes are projected to be significantly downregulated. Pathway analysis figures were created with Advaita iPathwayGuide.
