# Supplementary material for: Sertoli Cells Express Accommodation, Survival, and Immunoregulatory Factors When Exposed to Normal Human Serum
Source: Biomedicines. 2023 Jun 6;11(6):1650. doi: 10.3390/biomedicines11061650 (PMC10296330; doi:10.3390/biomedicines11061650)
Supplement: Supplementary file 1 [file biomedicines-11-01650-s001.zip › biomedicines-2422068-Figure S4.pdf]

A

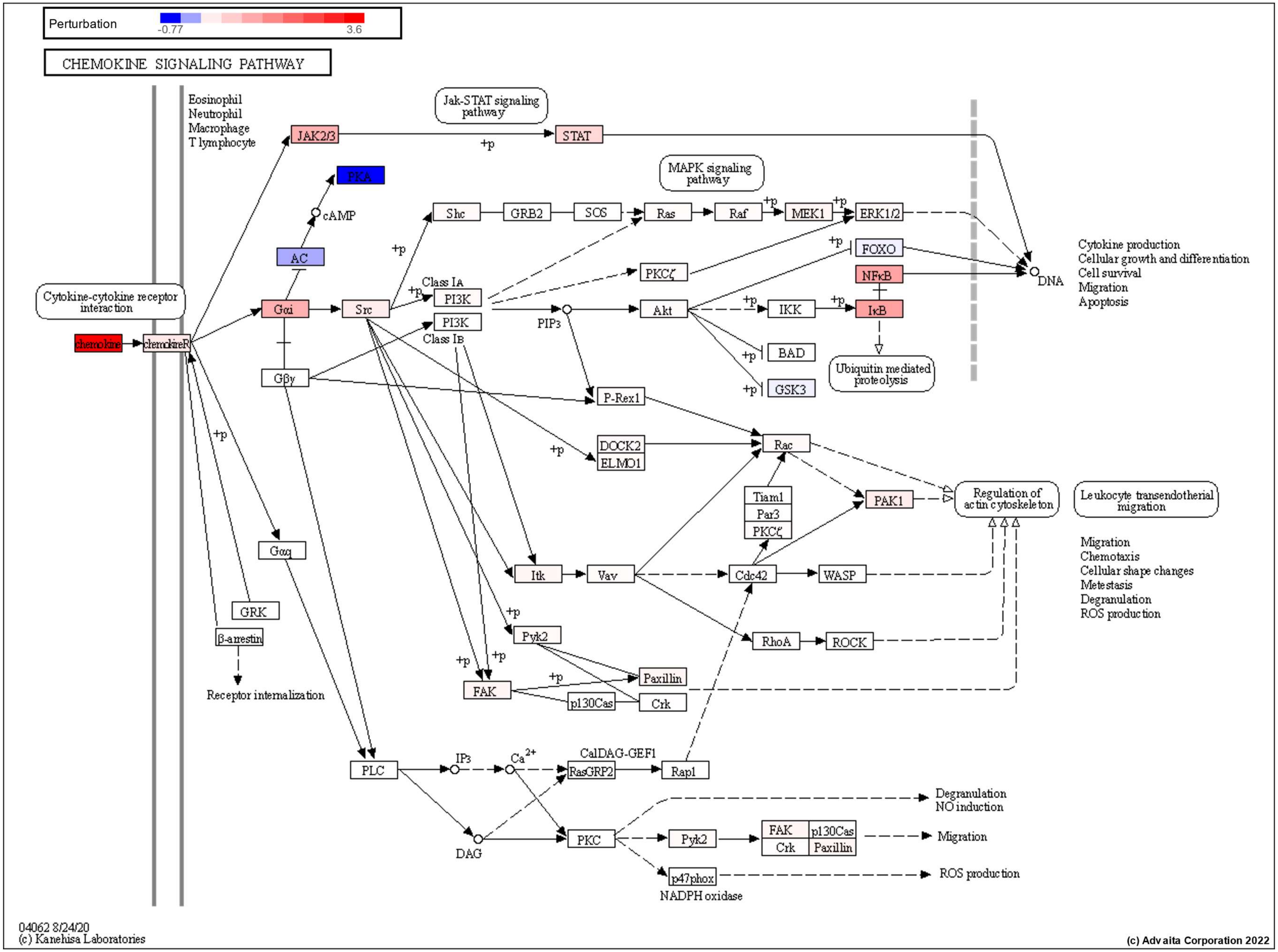
Perturbation
-0.77
3.6
CHEMOKINE SIGNALING PATHWAY
Eosinophil
Neutrophil
Macrophage
T lymphocyte
Jak-STAT signaling pathway
JAK2/3
+p
STAT
PKA
cAMP
AC
MAPK signaling pathway
Shc
GRB2
SOS
Ras
Raf
MEK1
ERK1/2
FOXO
NFκB
DNA
Cytokine production
Cellular growth and differentiation
Cell survival
Migration
Apoptosis
Cytokine-cytokine receptor interaction
Gαi
Src
Class IA
PI3K
PI3K
Class IB
PIP3
PKCζ
Akt
IKK
IκB
BAD
GSK3
Ubiquitin mediated proteolysis
chemokine
chemokineR
Gβγ
P-Rex1
DOCK2
ELMO1
Rac
Tiam1
Par3
PKCζ
PAK1
Regulation of actin cytoskeleton
Leukocyte transendothelial migration
Migration
Chemotaxis
Cellular shape changes
Metestasis
Degranulation
ROS production
Gαq
Itk
Vav
Cdc42
WASP
GRK
β-arrestin
Receptor internalization
Pyk2
RhoA
ROCK
Paxillin
FAK
p130Cas
Crk
PLC
IP3
Ca2+
CalDAG-GEF1
RasGRP2
Rap1
DAG
PKC
Pyk2
FAK
p130Cas
Crk
Paxillin
p47phox
NADPH oxidase
Degranulation
NO induction
Migration
ROS production
04062 8/24/20
(c) Kanehisa Laboratories
(c) Advaita Corporation 2022

B

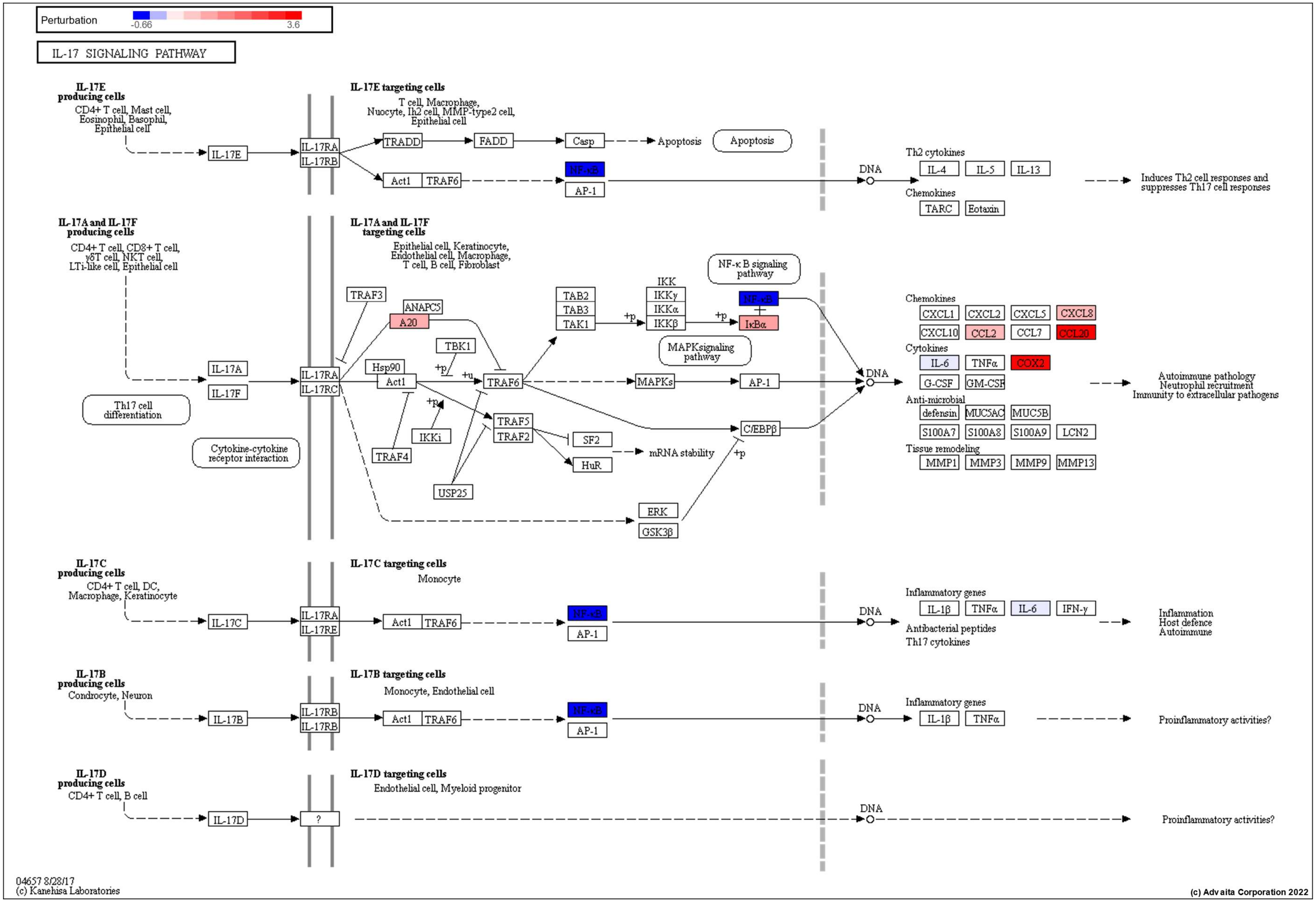

Perturbation
-0.66
3.6
IL-17 SIGNALING PATHWAY
IL-17E producing cells
CD4+ T cell, Mast cell, Eosinophil, Basophil, Epithelial cell
IL-17E
IL-17RA
IL-17RB
IL-17E targeting cells
T cell, Macrophage, Nuocyte, Ih2 cell, MMP-type2 cell, Epithelial cell
TRADD
FADD
Casp
Apoptosis
Apoptosis
Act1
TRAF6
NF-κB
AP-1
DNA
Th2 cytokines
IL-4
IL-5
IL-13
Chemokines
TARC
Eotaxin
Induces Th2 cell responses and suppresses Th17 cell responses
IL-17A and IL-17F producing cells
CD4+ T cell, CD8+ T cell, γδT cell, NKT cell, LTi-like cell, Epithelial cell
IL-17A
IL-17F
IL-17RA
IL-17RC
Th17 cell differentiation
Cytokine-cytokine receptor interaction
IL-17A and IL-17F targeting cells
Epithelial cell, Keratinocyte, Endothelial cell, Macrophage, T cell, B cell, Fibroblast
TRAF3
ANAPC5
A20
TBK1
Hsp90
Act1
+p
+u
TRAF6
IKKi
TRAF4
USP25
TRAF5
TRAF2
SF2
HuR
mRNA stability
TAB2
TAB3
TAK1
IKK
IKKγ
IKKα
IKKβ
+p
NF-κB signaling pathway
NF-κB
IκBα
MAPKsignaling pathway
MAPKs
AP-1
C/EBPβ
+p
ERK
GSK3β
DNA
Chemokines
CXCL1
CXCL2
CXCL5
CXCL8
CXCL10
CCL2
CCL7
CCL20
Cytokines
IL-6
TNFα
COX2
G-CSF
GM-CSF
Anti-microbial
defensin
MUC5AC
MUC5B
S100A7
S100A8
S100A9
LCN2
Tissue remodeling
MMP1
MMP3
MMP9
MMP13
Autoimmune pathology
Neutrophil recruitment
Immunity to extracellular pathogens
IL-17C producing cells
CD4+ T cell, DC, Macrophage, Keratinocyte
IL-17C
IL-17RA
IL-17RE
IL-17C targeting cells
Monocyte
Act1
TRAF6
NF-κB
AP-1
DNA
Inflammatory genes
IL-1β
TNFα
IL-6
IFN-γ
Antibacterial peptides
Th17 cytokines
Inflammation
Host defence
Autoimmune
IL-17B producing cells
Condrocyte, Neuron
IL-17B
IL-17RB
IL-17RB
IL-17B targeting cells
Monocyte, Endothelial cell
Act1
TRAF6
NF-κB
AP-1
DNA
Inflammatory genes
IL-1β
TNFα
Proinflammatory activities?
IL-17D producing cells
CD4+ T cell, B cell
IL-17D
?
IL-17D targeting cells
Endothelial cell, Myeloid progenitor
DNA
Proinflammatory activities?
04657 8/28/17
(c) Kanehisa Laboratories
(c) Advaita Corporation 2022

C

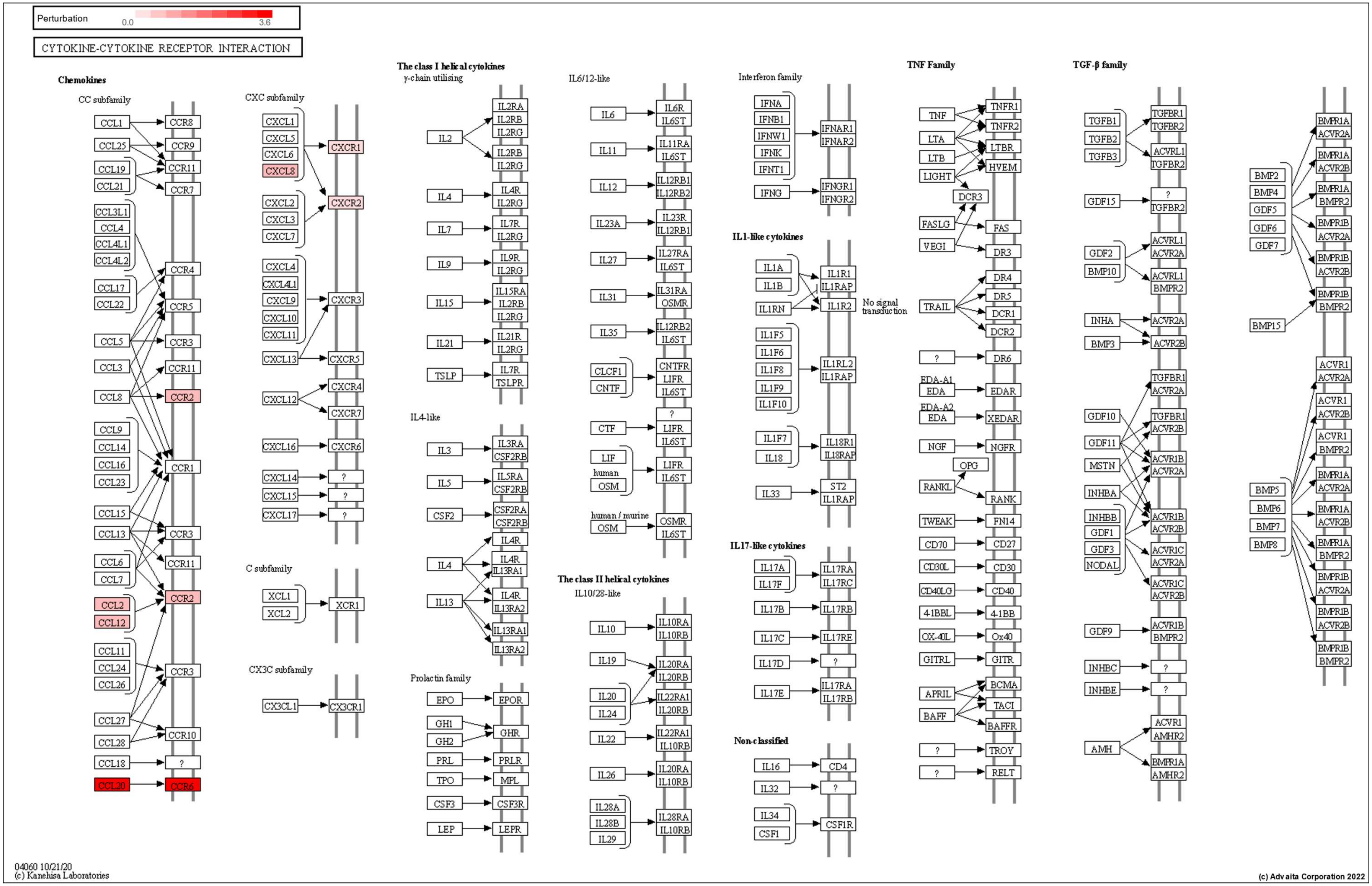

Perturbation
0.0
3.6
CYTOKINE-CYTOKINE RECEPTOR INTERACTION
Chemokines
CC subfamily
CCL1
CCL25
CCL19
CCL21
CCL3L1
CCL4
CCL4L1
CCL4L2
CCL17
CCL22
CCL5
CCL3
CCL8
CCL9
CCL14
CCL16
CCL23
CCL15
CCL13
CCL6
CCL7
CCL2
CCL12
CCL11
CCL24
CCL26
CCL27
CCL28
CCL18
CCL20
CCR8
CCR9
CCR11
CCR7
CCR4
CCR5
CCR3
CCR11
CCR2
CCR1
CCR3
CCR11
CCR2
CCR3
CCR10
?
CCR6
CXC subfamily
CXCL1
CXCL5
CXCL6
CXCL8
CXCL2
CXCL3
CXCL7
CXCL4
CXCL4L1
CXCL9
CXCL10
CXCL11
CXCL13
CXCL12
CXCL16
CXCL14
CXCL15
CXCL17
CXCR1
CXCR2
CXCR3
CXCR5
CXCR4
CXCR7
CXCR6
?
?
?
C subfamily
XCL1
XCL2
XCR1
CX3C subfamily
CX3CL1
CX3CR1
The class I helical cytokines
γ-chain utilising
IL2
IL2RA
IL2RB
IL2RG
IL2RB
IL2RG
IL4
IL4R
IL2RG
IL7
IL7R
IL2RG
IL9
IL9R
IL2RG
IL15
IL15RA
IL2RB
IL2RG
IL21
IL21R
IL2RG
TSLP
IL7R
TSLPR
IL4-like
IL3
IL3RA
CSF2RB
IL5
IL5RA
CSF2RB
CSF2
CSF2RA
CSF2RB
IL4
IL13
IL4R
IL4R
IL13RA1
IL4R
IL13RA2
IL13RA1
IL13RA2
Prolactin family
EPO
EPOR
GH1
GH2
GHR
PRL
PRLR
TPO
MPL
CSF3
CSF3R
LEP
LEPR
IL6/12-like
IL6
IL6R
IL6ST
IL11
IL11RA
IL6ST
IL12
IL12RB1
IL12RB2
IL23A
IL23R
IL12RB1
IL27
IL27RA
IL6ST
IL31
IL31RA
OSMR
IL35
IL12RB2
IL6ST
CLCF1
CNTF
CNTFR
LIFR
IL6ST
CTF
?
LIFR
IL6ST
LIF
human
OSM
LIFR
IL6ST
human / murine
OSM
OSMR
IL6ST
The class II helical cytokines
IL10/28-like
IL10
IL10RA
IL10RB
IL19
IL20RA
IL20RB
IL20
IL24
IL22RA1
IL20RB
IL22
IL22RA1
IL10RB
IL26
IL20RA
IL10RB
IL28A
IL28B
IL29
IL28RA
IL10RB
Interferon family
IFNA
IFNB1
IFNW1
IFNK
IFNT1
IFNAR1
IFNAR2
IFNG
IFNGR1
IFNGR2
IL1-like cytokines
IL1A
IL1B
IL1RN
IL1R1
IL1RAP
IL1R2
No signal transduction
IL1F5
IL1F6
IL1F8
IL1F9
IL1F10
IL1RL2
IL1RAP
IL1F7
IL18
IL18R1
IL18RAP
IL33
ST2
IL1RAP
IL17-like cytokines
IL17A
IL17F
IL17RA
IL17RC
IL17B
IL17RB
IL17C
IL17RE
IL17D
?
IL17E
IL17RA
IL17RB
Non-classified
IL16
CD4
IL32
?
IL34
CSF1
CSF1R
TNF Family
TNF
LTA
LTB
LIGHT
TNFR1
TNFR2
LTBR
HVEM
DCR3
FASLG
VEGI
FAS
DR3
TRAIL
DR4
DR5
DCR1
DCR2
?
DR6
EDA-A1
EDA
EDAR
EDA-A2
EDA
XEDAR
NGF
NGFR
OPG
RANKL
RANK
TWEAK
FN14
CD70
CD27
CD30L
CD30
CD40LG
CD40
4-1BBL
4-1BB
OX-40L
Ox40
GITRL
GITR
APRIL
BAFF
BCMA
TACI
BAFFR
?
TROY
?
RELT
TGF-β family
TGFB1
TGFB2
TGFB3
TGFBR1
TGFBR2
ACVRL1
TGFBR2
GDF15
?
TGFBR2
GDF2
BMP10
ACVRL1
ACVR2A
ACVRL1
BMPR2
INHA
BMP3
ACVR2A
ACVR2B
GDF10
GDF11
MSTN
INHBA
INHBB
GDF1
GDF3
NODAL
TGFBR1
ACVR2A
TGFBR1
ACVR2B
ACVR1B
ACVR2A
ACVR1B
ACVR2B
ACVR1C
ACVR2A
ACVR1C
ACVR2B
GDF9
ACVR1B
BMPR2
INHBC
?
INHBE
?
AMH
ACVR1
AMHR2
BMPR1A
AMHR2
BMP2
BMP4
GDF5
GDF6
GDF7
BMPR1A
ACVR2A
BMPR1A
ACVR2B
BMPR1A
BMPR2
BMPR1B
ACVR2A
BMPR1B
ACVR2B
BMPR1B
BMPR2
BMP15
ACVR1
ACVR2A
ACVR1
ACVR2B
ACVR1
BMPR2
BMP5
BMP6
BMP7
BMP8
BMPR1A
ACVR2A
BMPR1A
ACVR2B
BMPR1A
BMPR2
BMPR1B
ACVR2A
BMPR1B
ACVR2B
BMPR1B
BMPR2
04060 10/21/20
(c) Kanehisa Laboratories
(c) Advaita Corporation 2022

D

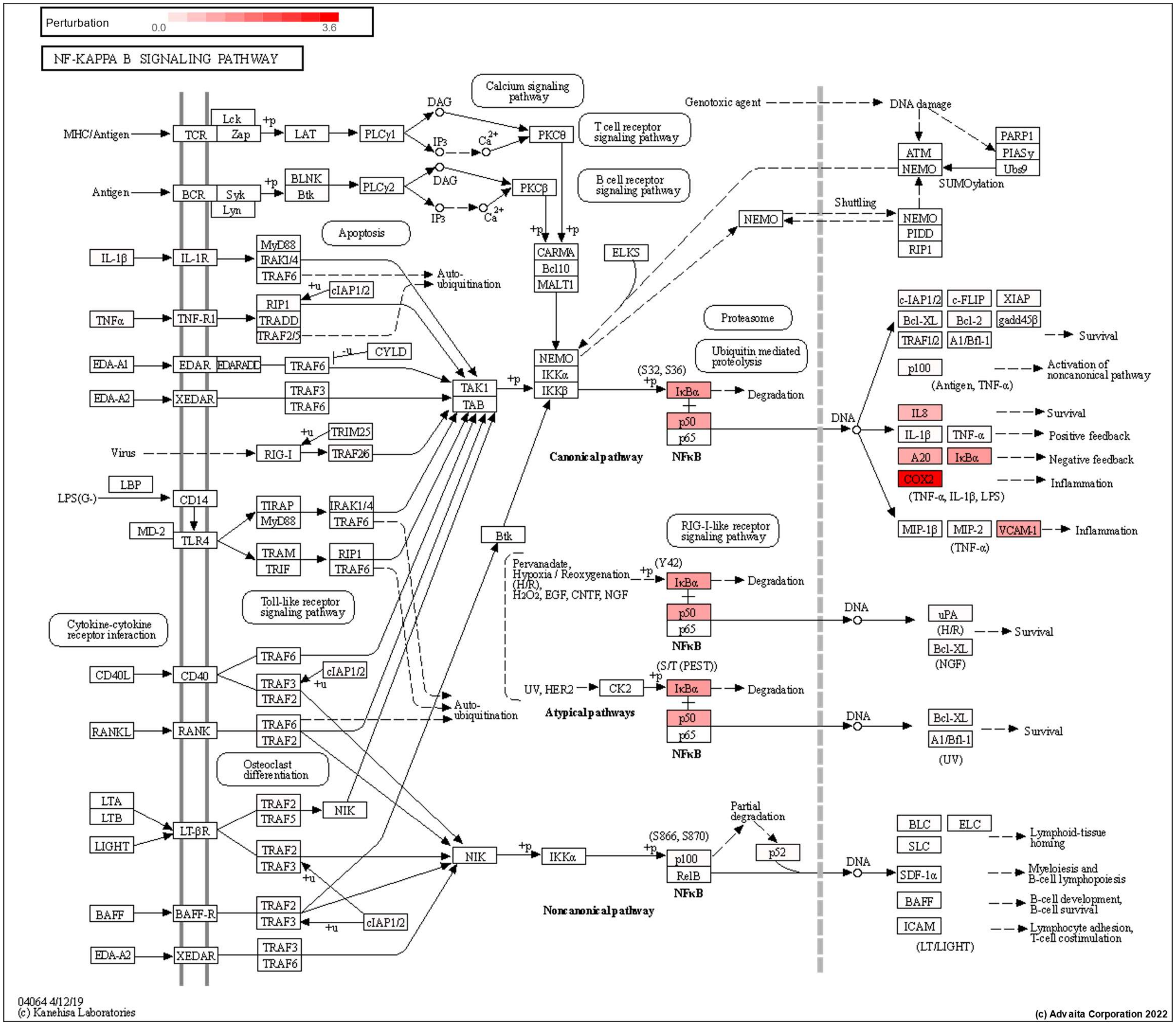

Perturbation
0.0
3.6
NF-KAPPA B SIGNALING PATHWAY
Calcium signaling pathway
T cell receptor signaling pathway
B cell receptor signaling pathway
Genotoxic agent
DNA damage
ATM
NEMO
PARP1
PIASy
Ubc9
SUMOylation
Shuttling
NEMO
PIDD
RIP1
MHC/Antigen
TCR
Lck
Zap
+p
LAT
PLCγ1
DAG
IP3
Ca2+
PKCθ
Antigen
BCR
Syk
Lyn
BLNK
Btk
PLCγ2
PKCβ
Apoptosis
CARMA
Bcl10
MALT1
ELKS
IL-1β
IL-1R
MyD88
IRAK1/4
TRAF6
Auto-ubiquitination
+u
cIAP1/2
TNFα
TNF-R1
RIP1
TRADD
TRAF2/5
EDA-A1
EDAR
EDARADD
TRAF6
-u
CYLD
EDA-A2
XEDAR
TRAF3
TRAF6
TAK1
TAB
NEMO
IKKα
IKKβ
Proteasome
Ubiquitin mediated proteolysis
(S32, S36)
IκBα
Degradation
p50
p65
NFκB
Canonical pathway
Virus
RIG-I
TRIM25
TRAF2/6
LBP
LPS(G-)
CD14
MD-2
TLR4
TIRAP
MyD88
IRAK1/4
TRAF6
TRAM
TRIF
RIP1
TRAF6
Toll-like receptor signaling pathway
Cytokine-cytokine receptor interaction
Btk
Pervanadate, Hypoxia / Reoxygenation (H/R), H2O2, EGF, CNTF, NGF
(Y42)
RIG-I-like receptor signaling pathway
UV, HER2
CK2
(S/T (PEST))
Atypical pathways
CD40L
CD40
TRAF6
TRAF3
TRAF2
RANKL
RANK
Osteoclast differentiation
LTA
LTB
LIGHT
LT-βR
TRAF2
TRAF5
NIK
IKKα
(S866, S870)
p100
RelB
Partial degradation
p52
Noncanonical pathway
BAFF
BAFF-R
EDA-A2
XEDAR
DNA
c-IAP1/2
c-FLIP
XIAP
Bcl-XL
Bcl-2
gadd45β
TRAF1/2
A1/Bfl-1
Survival
p100
Activation of noncanonical pathway
(Antigen, TNF-α)
IL8
IL-1β
TNF-α
A20
IκBα
COX2
(TNF-α, IL-1β, LPS)
Positive feedback
Negative feedback
Inflammation
MIP-1β
MIP-2
VCAM-1
(TNF-α)
uPA
(H/R)
Bcl-XL
(NGF)
Bcl-XL
A1/Bfl-1
(UV)
BLC
ELC
SLC
SDF-1α
BAFF
ICAM
(LT/LIGHT)
Lymphoid-tissue homing
Myeloiesis and B-cell lymphopoiesis
B-cell development, B-cell survival
Lymphocyte adhesion, T-cell costimulation
04064 4/12/19
(c) Kanehisa Laboratories
(c) Advaita Corporation 2022

E

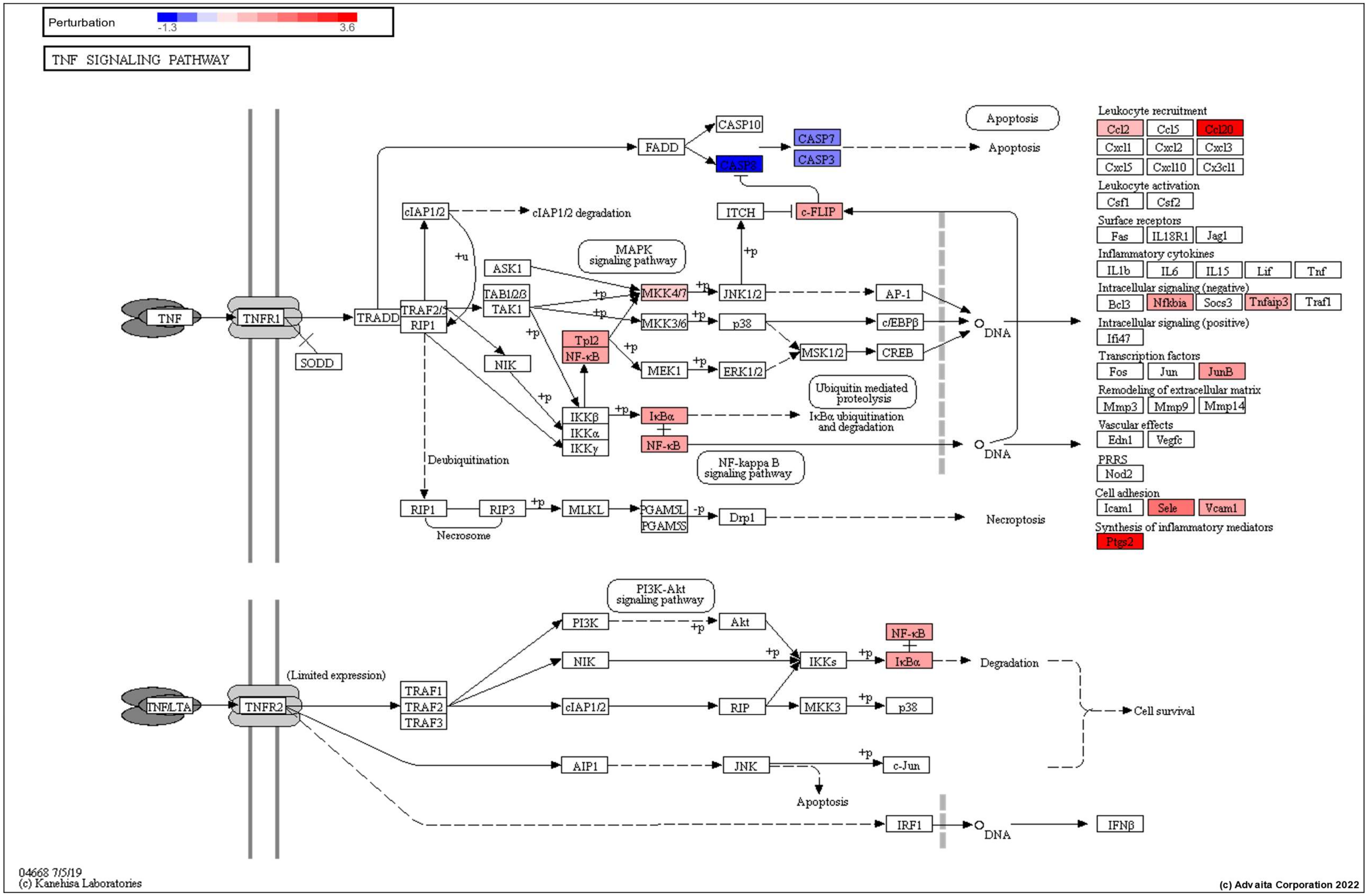

Figure S4. Cytokine signaling pathways. Cytokine signaling pathways in NPSC affected by NHS include (A) chemokine signaling, (B) IL-17 signaling, (C) cytokine-cytokine receptor interactions, (D) NF-κB signaling, and (E) TNF signaling pathways. Pathway perturbations are represented. Red highlighted genes are projected to be significantly upregulated while blue highlighted genes are projected to be significantly downregulated. Pathway analysis figures were created with Advaita iPathwayGuide.
