# Supplementary material for: Sertoli Cells Express Accommodation, Survival, and Immunoregulatory Factors When Exposed to Normal Human Serum
Source: Biomedicines. 2023 Jun 6;11(6):1650. doi: 10.3390/biomedicines11061650 (PMC10296330; doi:10.3390/biomedicines11061650)
Supplement: Supplementary file 1 [file biomedicines-11-01650-s001.zip › biomedicines-2422068-Figure S5.pdf]

A

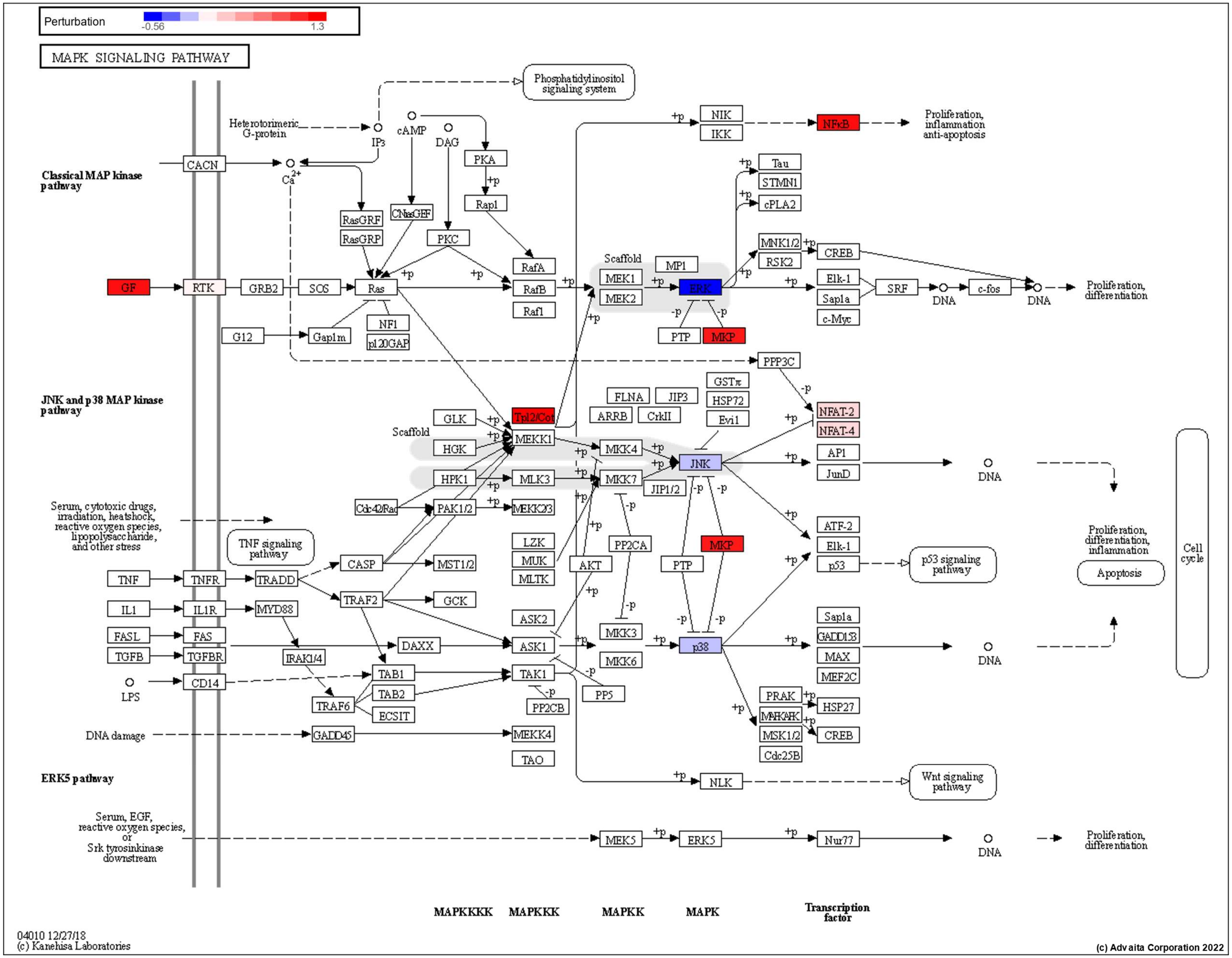
Perturbation
-0.56
1.3
MAPK SIGNALING PATHWAY
Phosphatidylinositol signaling system
Heterotrimeric G-protein
IP3
cAMP
DAG
CACN
Ca2+
PKA
Rap1
Classical MAP kinase pathway
RasGRF
RasGRP
CNrasGEF
PKC
RafA
RafB
Raf1
GF
RTK
GRB2
SOS
Ras
NF1
p120GAP
G12
Gap1m
Scaffold
MEK1
MEK2
MP1
ERK
PTP
MKP
NIK
IKK
NFκB
Proliferation, inflammation anti-apoptosis
Tau
STMN1
cPLA2
MNK1/2
RSK2
CREB
Elk-1
Sap1a
c-Myc
SRF
c-fos
DNA
Proliferation, differentiation
PPP3C
NFAT-2
NFAT-4
JNK and p38 MAP kinase pathway
GLK
HGK
HPK1
Cdc42/Rac
PAK1/2
Tpl2/Cot
MEKK1
MLK3
MEKK2/3
LZK
MUK
MLTK
FLNA
JIP3
ARRB
CrkII
GSTπ
HSP72
Evi1
MKK4
MKK7
JIP1/2
JNK
AP1
JunD
ATF-2
p53
p53 signaling pathway
Serum, cytotoxic drugs, irradiation, heatshock, reactive oxygen species, lipopolysaccharide, and other stress
TNF signaling pathway
TNF
TNFR
TRADD
CASP
MST1/2
TRAF2
GCK
IL1
IL1R
MYD88
FASL
FAS
DAXX
TGFB
TGFBR
IRAK1/4
LPS
CD14
TAB1
TAB2
TRAF6
ECSIT
AKT
PP2CA
ASK2
ASK1
TAK1
PP2CB
MKK3
MKK6
PP5
p38
GADD153
MAX
MEF2C
PRAK
MAPKAPK
MSK1/2
Cdc25B
HSP27
Proliferation, differentiation, inflammation
Apoptosis
Cell cycle
DNA damage
GADD45
MEKK4
TAO
NLK
Wnt signaling pathway
ERK5 pathway
Serum, EGF, reactive oxygen species, or Srk tyrosinkinase downstream
MEK5
ERK5
Nur77
+p
-p
MAPKKKK
MAPKKK
MAPKK
MAPK
Transcription factor
04010 12/27/18
(c) Kanehisa Laboratories
(c) Advaita Corporation 2022

**B**

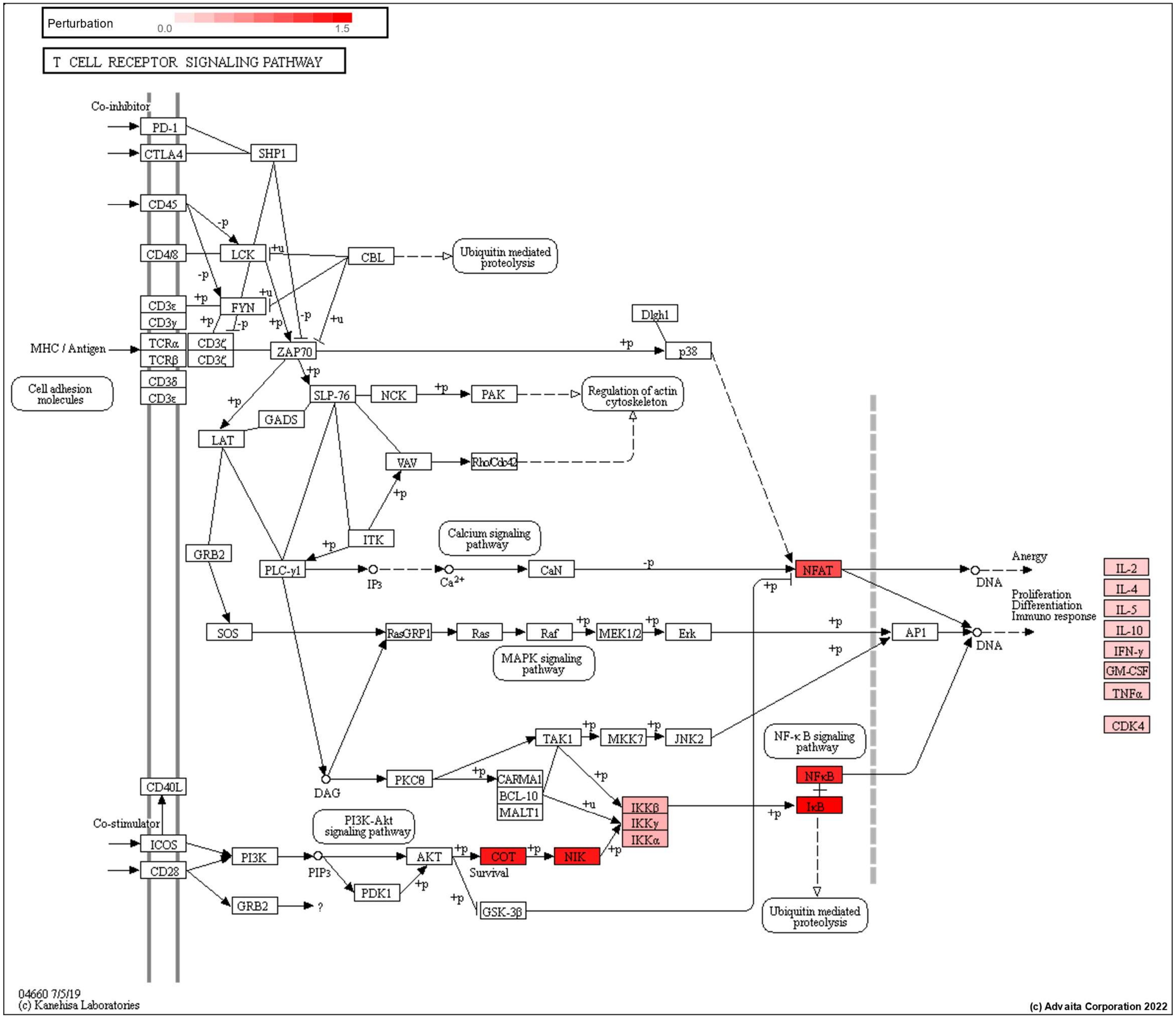
Perturbation
0.0
1.5
T CELL RECEPTOR SIGNALING PATHWAY
Co-inhibitor
PD-1
CTLA4
SHP1
CD45
CD4/8
LCK
CBL
Ubiquitin mediated proteolysis
CD3ε
CD3γ
FYN
Dlgh1
MHC / Antigen
TCRα
TCRβ
CD3ζ
ZAP70
p38
Cell adhesion molecules
CD3δ
SLP-76
NCK
PAK
Regulation of actin cytoskeleton
GADS
LAT
VAV
RhoCdc42
ITK
Calcium signaling pathway
GRB2
PLC-γ1
IP3
Ca2+
CaN
NFAT
DNA
Anergy
Proliferation
Differentiation
Immuno response
IL-2
IL-4
IL-5
IL-10
IFN-γ
GM-CSF
TNFα
CDK4
SOS
RasGRP1
Ras
Raf
MEK1/2
Erk
AP1
MAPK signaling pathway
TAK1
MKK7
JNK2
NF-κB signaling pathway
CD40L
DAG
PKCθ
CARMA1
BCL-10
MALT1
IKKβ
IKKγ
IKKα
NFκB
IκB
Co-stimulator
PI3K-Akt signaling pathway
ICOS
CD28
PI3K
PIP3
AKT
COT
NIK
Survival
PDK1
GRB2
GSK-3β
04660 7/5/19
(c) Kanehisa Laboratories
(c) Advaita Corporation 2022

C

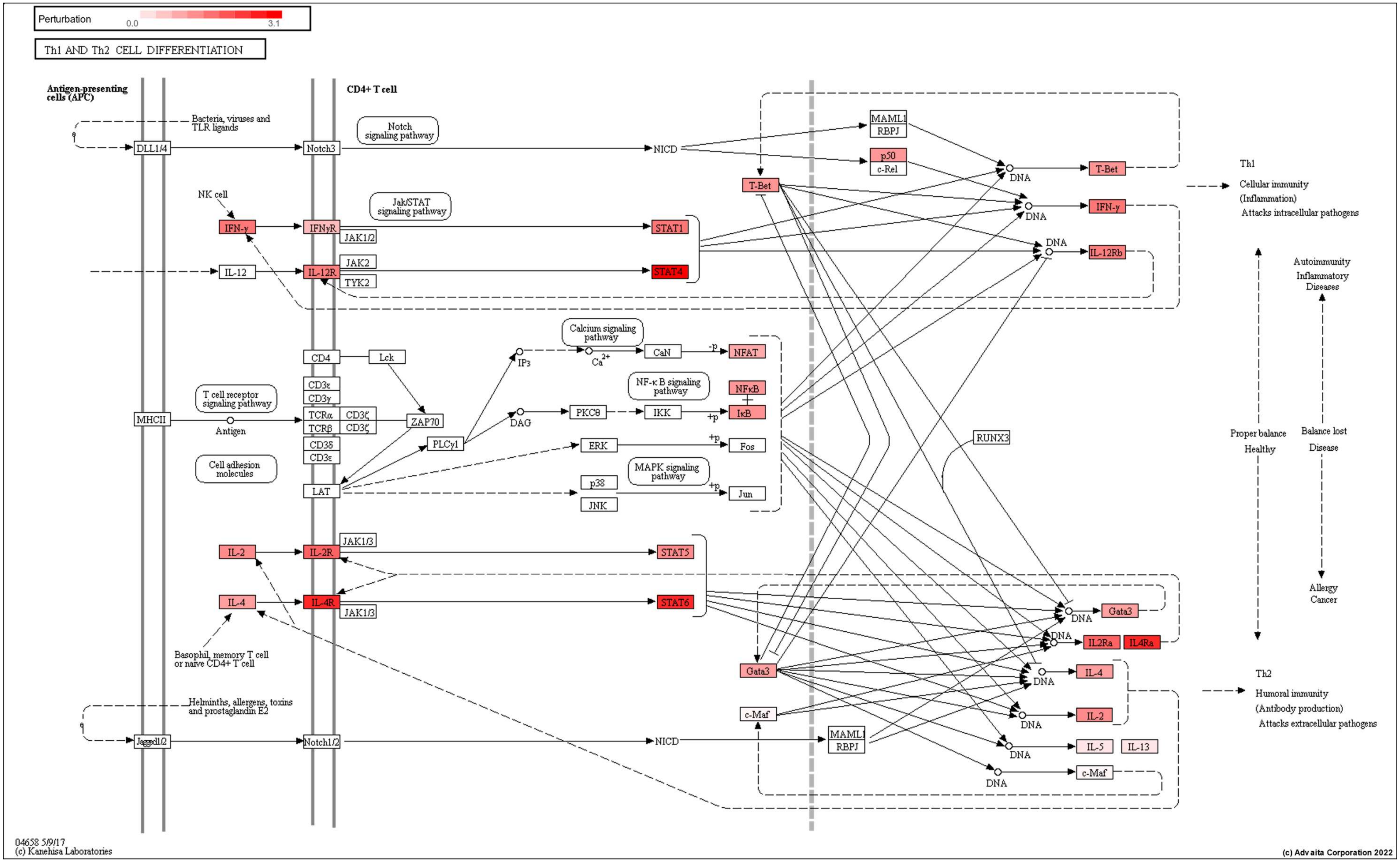

Perturbation
0.0
3.1
Th1 AND Th2 CELL DIFFERENTIATION
Antigen-presenting cells (APC)
CD4+ T cell
Bacteria, viruses and TLR ligands
DLL1/4
Notch3
Notch signaling pathway
NICD
MAML1
RBPJ
p50
c-Rel
DNA
T-Bet
Th1
Cellular immunity
(Inflammation)
Attacks intracellular pathogens
NK cell
IFN-γ
IFNγR
JAK1/2
Jak/STAT signaling pathway
STAT1
IL-12
IL-12R
JAK2
TYK2
STAT4
IL-12Rb
Autoimmunity
Inflammatory
Diseases
CD4
Lck
Calcium signaling pathway
IP3
Ca2+
CaN
NFAT
NF-κB signaling pathway
NFκB
IκB
CD3ε
CD3γ
T cell receptor signaling pathway
MHCII
Antigen
TCRα
TCRβ
CD3ζ
ZAP70
DAG
PKCθ
IKK
PLCγ1
ERK
Fos
Cell adhesion molecules
CD3δ
LAT
p38
JNK
MAPK signaling pathway
Jun
RUNX3
Proper balance
Healthy
Balance lost
Disease
IL-2
IL-2R
JAK1/3
STAT5
IL-4
IL-4R
STAT6
Gata3
IL2Ra
IL4Ra
Allergy
Cancer
Basophil, memory T cell or naive CD4+ T cell
Th2
Humoral immunity
(Antibody production)
Attacks extracellular pathogens
Helminths, allergens, toxins and prostaglandin E2
c-Maf
Jagged1/2
Notch1/2
IL-5
IL-13
04658 5/9/17
(c) Kanehisa Laboratories
(c) Advaita Corporation 2022

**D**

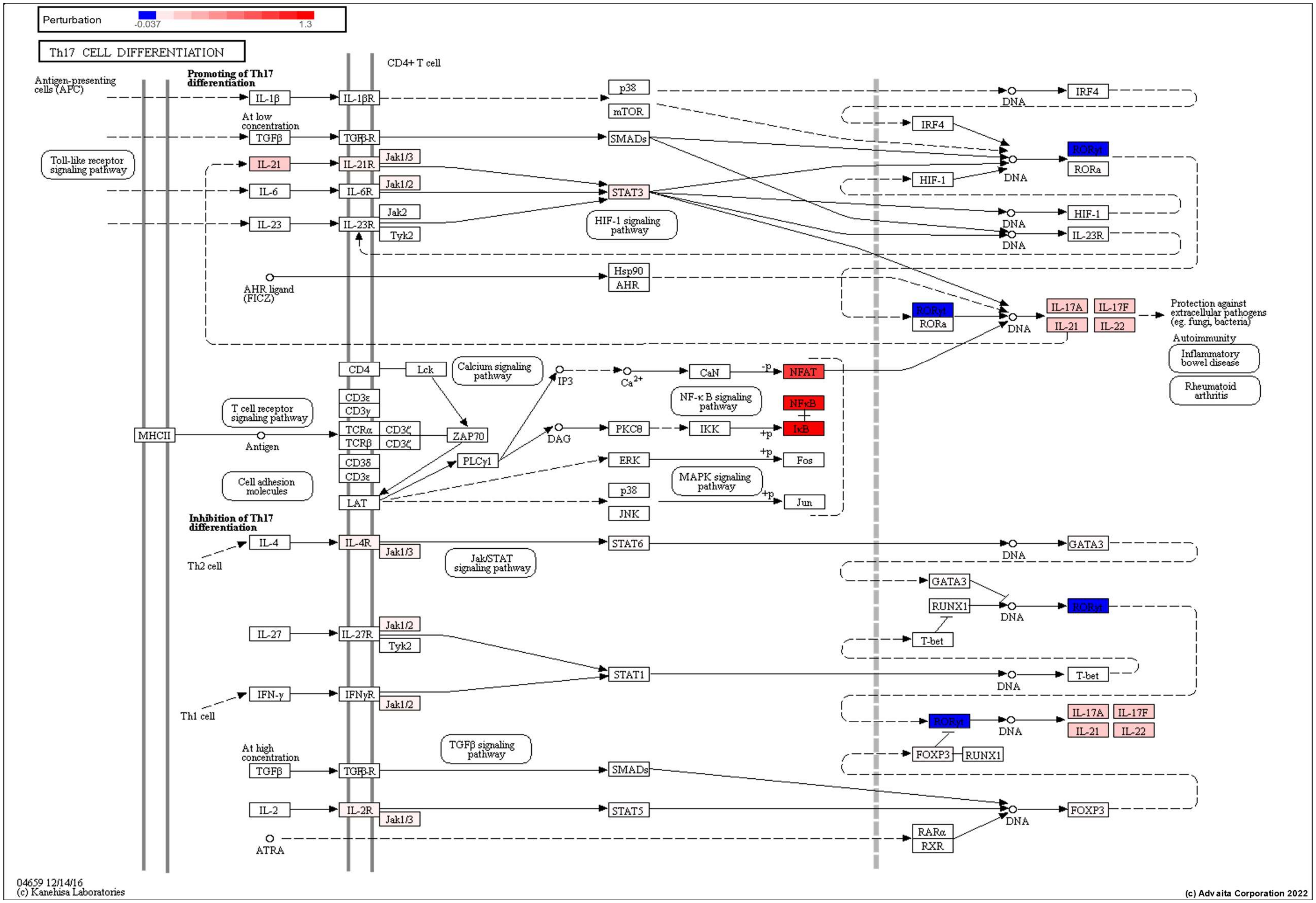


**Figure S5.** T cell regulation. T cell regulatory pathways in NPSC affected by NHS include (A) MAPK signaling, (B) TCR signaling, (C) Th1/Th2 cell activation, and (D) Th17 cell activation. Pathway perturbations are represented. Red highlighted genes are projected to be significantly upregulated while blue highlighted genes are projected to be significantly downregulated. Pathway analysis figures were created with Advaita iPathwayGuide.
