# Supplementary material for: Sertoli Cells Express Accommodation, Survival, and Immunoregulatory Factors When Exposed to Normal Human Serum
Source: Biomedicines. 2023 Jun 6;11(6):1650. doi: 10.3390/biomedicines11061650 (PMC10296330; doi:10.3390/biomedicines11061650)
Supplement: Supplementary file 1 [file biomedicines-11-01650-s001.zip › biomedicines-2422068-supplementary tables.pdf]

Supplementary Materials

Table S1. Genes of interest PCR primers.

Gene	Forward Primer (5’ → 3’)	Reverse Primer (5’ → 3’)
C6	GAGCTTGCTCCCATCACAGA	TGGCTGCGTATTCTCGGAAA
CCL2	CCAGGACTCCATAAGCCACC	AATGTGCCCAAGTCTCCGTT
CCL20	TCGCCAATGAAGCTTGTGAC	CCAATTCCATAGCAGGGAGCA
CXCL8	GCAGAGCTCACAAGCTCCTA	AAGTCATGTTGACACGGGCT
EGR1	CCTTCGGCTCCTTTCCACAT	GGGAAAAGACTCTGCGGTCA
GAPDH*	CCTCCCCGTTCGACAGACA	GATGCGGCCAAATCCGTT
KERA	GGAGCCCTGAGTCAACTGAA	GCAGGTCAAGAAGGGTCAGG
LYZ	GCAAGACACCCAAAGCAGTT	ACCCCGAATGTACTGCGAGA
NFKB1	CACGCCAATGCCCTTTTTGA	GTTACCCAAGCGGTCCAGAA
NFKBIA	AGACTCGTTCCTGCACTTGG	TCTCGGAGCTCAGGATCACA
PTGS2	CAGAAGCGAGGACCAGCTTT	CAGCCAGATTGTGGCGTACA
SUSD4	GAGACTCTTGGCCGTGATCC	AGCTGGAATCCGTCTTGGCA
TNFAIP3	AAGTGAGGAAGCTTGTGGCA	AATCCTCAGTCCAATTCCGGG

*Endogenous control

Table S2. qPCR quantification of GOI between baseline and complement NPSC.

Gene	Baseline	Complement	-log2(FC)	p-value
C6	1.008	1.574	3.776	0.072983
CCL2	1.008	6.480	4.794	0.036041
CCL20	1.014	25.02	10.97	0.000499
CXCL8	1.001	4.466	5.165	0.027880
EGR1	1.005	6.944	12.56	0.000166
KERA	1.002	1.271	1.011	0.496254
LYZ	1.010	0.054	19.30	0.000002
NFKB1	1.001	2.982	11.46	0.000356
NFKBIA	1.005	6.389	12.57	0.000165
PTGS2	1.009	23.73	7.108	0.007250
SUSD4	1.006	1.116	1.639	0.321186
TNFAIP3	1.005	5.478	7.444	0.005741

Table S3. Genes of interest.

Gene	Gene Name	Function
C6	Complement component 6	Part of the MAC
CCL2	C-C motif chemokine ligand 2	Cytokine involved in chemotaxis of monocytes, basophils, memory T cells, and dendritic cells; receptors include CCR2 and CCR14
CCL20	C-C motif chemokine ligand 20	Antimicrobial cytokine; immune regulation; inflammation; chemotaxis of dendritic cells, effector lymphocytes, and memory lymphocytes; CCR6 is cognate receptor
CXCL8	C-X-C motif chemokine ligand 8 (i.e., IL-8)	Chemotactic factor; inflammation; angiogenesis; increases blood vessel permeability; receptors include CXCR1 and CXCR2
EGR1	Early growth response 1	Transcription factor for differentiation and mitogenesis genes
KERA	Keratocan	Corneal transparency and structure
LYZ	Lysozyme	Antimicrobial substance; cleaves β[1-4]glycosidic linkages between N-acetylmuramic acid and N-acetylglucosamine of peptidoglycans that make up bacterial cell walls
NFKB1	Nuclear factor kappa B subunit 1	Transcription regulator for genes involved in many biological processes; regulator of immediate viral responses; associated with inflammation
NFKBIA	NFKB inhibitor alpha (i.e., IKBA)	Inhibits NFκB function and translocation to nucleus
PTGS2	Prostaglandin-endoperoxide synthase 2 (i.e., cyclooxygenase)	Prostaglandin biosynthesis in inflammation and mitogenesis
SUSD4	Sushi domain containing 4	Membrane-bound inhibition of alternative and classical complement activation
TNFAIP3	Tumor necrosis factor alpha induced protein 3 (i.e., A20)	Inhibits NFκB function; inhibits TNF-mediated apoptosis; associated with transplant accommodation and survival

Data retrieved from NCBI Gene Database [30].
